# Regulation of LRRK2 Expression Points to a Functional Role in Human Monocyte Maturation

**DOI:** 10.1371/journal.pone.0021519

**Published:** 2011-06-27

**Authors:** Jonathan Thévenet, Rosanna Pescini Gobert, Robertus Hooft van Huijsduijnen, Christoph Wiessner, Yves Jean Sagot

**Affiliations:** 1 Pharmacology Parkinson's Disease, TA-NDD Research, Merck-Serono SA, Geneva, Switzerland; 2 Multiple Sclerosis Drug Discovery, TA-NDD Research, Merck-Serono SA, Geneva, Switzerland; University of Brescia, Italy

## Abstract

Genetic variants of Leucine-Rich Repeat Kinase 2 (LRRK2) are associated with a significantly enhanced risk for Parkinson disease, the second most common human neurodegenerative disorder. Despite major efforts, our understanding of LRRK2 biological function and regulation remains rudimentary. In the present study we analyze LRRK2 mRNA and protein expression in sub-populations of human peripheral blood mononuclear cells (PBMCs). LRRK2 mRNA and protein was found in circulating CD19^+^ B cells and in CD14^+^ monocytes, whereas CD4^+^ and CD8^+^ T cells were devoid of LRRK2 mRNA. Within CD14^+^ cells the CD14^+^CD16^+^ sub-population of monocytes exhibited high levels of LRRK2 protein, in contrast to CD14^+^CD16^-^ cells. However both populations expressed LRRK2 mRNA. As CD14^+^CD16^+^ cells represent a more mature subset of monocytes, we monitored LRRK2 expression after *in vitro* treatment with various stress factors known to induce monocyte activation. We found that IFN-γ in particular robustly increased LRRK2 mRNA and protein levels in monocytes concomitant with a shift of CD14^+^CD16^−^ cells towards CD14^+^CD16^+^cells. Interestingly, the recently described LRRK2 inhibitor IN-1 attenuated this shift towards CD14^+^CD16^+^ after IFN-γ stimulation. Based on these findings we speculate that LRRK2 might have a role in monocyte maturation. Our results provide further evidence for the emerging role of LRRK2 in immune cells and regulation at the transcriptional and translational level. Our data might also reflect an involvement of peripheral and brain immune cells in the disease course of PD, in line with increasing awareness of the role of the immune system in PD.

## Introduction

Parkinson's disease (PD) is the second most common neurodegenerative disease affecting 1.5% of the population over 50 years [1]. Recent studies have linked several genes with PD [1], although the majority of PD cases is sporadic. Among associated genes, Leucine-Rich Repeat Kinase 2 (LRRK2 alias Dardarin) stands out since in some populations up to 30% of all PD patients carry the G2019S mutation [2]. LRRK2 is a large and complex 2,527 amino-acid protein that contains a ROC-COR domain with GTPase activity and a kinase domain with homology to MAPKKKs. Overall, biological functions of LRRK2 remain largely unknown, and the identification of physiological substrates remains controversial [3]. Nevertheless, there is consensus that LRRK2 multimerizes, auto-phosphorylates, and exists predominantly in a dimeric conformation when active [4].

Disease-associated mutations are localized in the ROC-COR and kinase domains, but not all result in modification of GTPase or kinase activities, leaving the pathogenic mechanism of such mutations unresolved [3]. It has been reported that the LRRK2 I2020T mutation is associated with enhanced intracellular degradation [5]. Studies performed in *Caenorhabditis elegans* or *Drosophila*, as well as recent studies using bacterial artificial chromosome (BAC) transgenic mice suggest that mutations in LRRK2 disturb the dopaminergic system, i.e. decrease dopamine release and cause motor deficits [6]–[7]. Since LRRK2 deficient [8] or LRRK2 wt over-expressing [9] transgenic mice do not present severe clinical neurological symptoms it seems likely that pathological mutations are not associated with a simple gain or loss of kinase or GTPase activity (for review [10]).

In general, these studies rely on artificially over-expressing or knocking down LRRK2 expression. Since LRRK2 is thought to be a stress kinase [11], and therefore can be expected to be tightly regulated, we believe it is important to study its function and regulation at endogenous levels. Due to its pathophysiological role in PD, the major focus to date was to study LRRK2 function in the brain. However, mRNA analysis revealed that LRRK2 is also highly expressed in peripheral organs such as kidney, lung, spleen and peripheral blood mononuclear cells [12]–[13]. The expression of LRRK2 in immune cells [14]–[15] supports the idea that LRRK2 could play a role in B cell development.

In contrast to neuronal cells, human PBMCs are easily accessible and present a valuable source for studying LRRK2 biology. The current work describes the characterization of a human PBMC sub-population expressing LRRK2 and the regulation and stabilization of LRRK2 by IFN-γ at the mRNA and protein level. Finally, experiments using LRRK2 kinase inhibitors suggest that LRRK2 may play an important role in monocyte responses to IFN-γ. From a clinical perspective our data suggest that hPBMC and more specifically monocytes derived from hPBMCs might yield biomarkers for therapeutic LRRK2 inhibitors.

## Materials and Methods

### Reagents

Different inducers of cellular stress and cytokines were used in this study: recombinant Human IFN-γ, TNF-α, IL-1β, IGF-1 (R&D Systems GmbH, Minneapolis, MN), LPS (from *E. coli* O111:B4, Sigma-Aldrich, St. Louis, MO), and H_2_O_2_ (Sigma-Aldrich, St. Louis, MO).

Several LRRK2 inhibitors were used: H1152 (Toronto Research Chemicals Inc., Ontario Canada), Sunitinib (Sellek Chemicals, Texas, USA), K252a (Sigma-Aldrich, St. Louis, MO), Y27632 (Tocris Bioscience, Bristol, UK) and IN-1 (Generous gift from Dr. D. Alessi, College of Life Science, University of Dundee, Dundee, UK).

### Antibodies

Three different antibodies against LRRK2 were used in this study. Rabbit polyclonal antibody to LRRK2 (ref. ab60937) was purchased from Abcam (Cambridge, UK), rabbit polyclonal antibody to LRRK2 (AT106) from Alexis Biochemicals (Enzo Life Sciences Inc., Plymouth Meeting, PA) and rabbit monoclonal antibody to LRRK2 (clone MJFF3-c69-6) from Epitomics Inc.(Burlingame, CA).

Mouse monoclonal antibody anti-Actin (clone C4) was from Merck-Millipore (DE). Mouse monoclonal antibody anti-GAPDH (6C5) was from HyTest Ltd (Turku, FI). Mouse monoclonal antibody anti-Hsp70 (Hsp72, C92F3A-5) was from Stressgen® (Enzo Life Sciences Inc., Plymouth Meeting, PA).

For chemiluminescence Western blotting, goat polyclonal antibody anti-Rabbit IgG/HRP was from Bio-Rad Laboratories (Hercules, CA) and goat polyclonal antibody anti-Mouse IgG/ HRP from DakoCytomation (Carpinteria, CA). IRDye® 680 donkey polyclonal antibody anti-mouse IgG and IRDye® 800CW donkey polyclonal anti-Rabbit IgG were from LI-COR Biosciences (Lincoln, NE).

For FACS analysis, mouse anti-human CD3 FITC, CD4 FITC or PE, CD8 PE, CD14 FITC, PE, PerCP-Cy5.5 or APC, CD16 FITC, CD19 FITC, HLA-DR APC, CD40 FITC, anti-CD54 PE, CD62L FITC, CD68 PE, CD71 APC, CD80 FITC, CD83 PE, CD103 FITC, CD206 PE and isotypes control (FITC mouse IgG1k, PE mouse IgG2ak and APC mouse IgG2ak) were purchased from BD Biosciences (Franklin Lakes, NJ). Mouse anti-human CD16 APC was from InVitrogen Ltd (Paisley, UK).

### Isolation of human peripheral blood mononuclear cells

Buffy-coats were obtained from anonymous blood donors via Geneva Transfusion Center (HUG, Geneva, Switzerland). The present study was approved by Merck Serono institutional committee of scientists, and by a Merck Serono Biosafety committee in charge to guarantee the correct use of the human material, accordingly to ethical and safety rules.

Human peripheral blood mononuclear cells (PBMC) were prepared from Buffy-coats.

Briefly, the blood sample was first diluted twice in sterile PBS and overlaid onto Ficoll-Paque PLUS (GE Healthcare, Piscataway, NJ) and spun at 400 g for 20 minutes at room temperature without brake. PBMC were collected at the plasma/Ficoll interface and washed third with PBS. Quality of the preparation was assessed by cell counting using a Coulter ACT-5 machine (Beckman Coulter Inv., Fullerton, CA).

### Isolation of human PBMC sub-populations

Depending on the type of experiment, human PBMC sub-populations were isolated from fresh PBMCs using cell sorting or immuno-magnetic beads (MACS™, Miltenyi Biotec). After MACS separation, cell subpopulations were stained for flow-cytometric analysis using combinations of fluorochrome-conjugated MAbs.

#### Isolation by FACS

Human PBMC were incubated with normal mouse IgG (InVitrogen Ltd, Paisley, UK) for 15 min at 4°C to block Fc-mediated unspecific binding and labeled with appropriate mouse monoclonal antibody anti-human CD for 1 h at 4°C. Washing were performed with PBS, 0.1% BSA (Albumin from Bovine Serum, Sigma-Aldrich, St. Louis, MO) and 0.01% NaN_3_ (sodium azide, Sigma-Aldrich, St. Louis, MO). Cells were sorted using a FACSAria cell sorter (Becton Dickinson, San Jose, CA).

#### Isolation with immunomagnetic beads

CD14^+^CD16^±^ monocytes were purified from human PBMC by negative selection using human monocytes enrichment kit without CD16 depletion (19058, StemCell Technologies, Köln, DE).

Monocyte sub-population CD16^-^ was obtained by two different ways:

- Negative selection of CD14^+^CD16^±^ monocytes using human monocytes enrichment kit (19059, StemCell Technologies, Köln, DE).
- Negative selection of CD14^+^CD16^±^ monocytes using APC selection kit with APC anti-human CD16 antibody (18451, StemCell Technologies, Köln, DE).

CD16^+^ monocytes were obtained by positive selection.

Sub-populations obtained with StemCell methods were analyzed by flow cytometer for CD14 and CD16 expression, in order to evaluate their purity.

### Flow cytometry analysis

Cells were incubated with normal mouse IgG (Invitrogen Ltd, Paisley, UK) for 15 min at 4°C to block Fc-mediated non-specific binding and labeled with appropriate conjugated antibodies for 1 h at 4°C. Washing was performed with PBS, 0.1% BSA (Albumin from Bovine Serum, Sigma-Aldrich, St. Louis, MO) and 0.01% NaN_3_ (sodium azide, Sigma-Aldrich, St. Louis, MO). Staining with anti-CD68 PE was performed after fixation and permeabilization with BD Cytofix/CytopermTM kit ((BD). Cells were analyzed using a FACSCalibur flow cytometer (Becton Dickinson, San Jose, CA). One hundred thousand events were acquired for each sample. Data were analyzed using FlowJo 7.6 software (Tree Star, Ashland, OR) and results were expressed as a percentage of total cells.

### Cells cultures and treatment

Two millions of freshly prepared hPBMC or monocytes CD14^+^CD16^±^ subpopulations were plated immediately in 2 mL of RPMI 1640 medium (InVitrogen Ltd, Paisley, UK) supplemented with 10% FBS (HyClone Laboratories), penicillin/streptomycin (InVitrogen Ltd, Paisley, UK), NEAA (InVitrogen Ltd, Paisley, UK), 1 mM Sodium Pyruvate (InVitrogen Ltd, Paisley, UK) in 6 wells cell culture cluster (Costar, ref 3516) at 37°C in a humidified atmosphere containing 5% CO_2_. In some experiment (specified in text) cells were kept over-night at 4°C in PBS before plating. Human recombinant IFN-γ, TNF-α, IL-1β, IGF-1, IL-6, LPS, H_2_O_2_ were diluted in PBS and directly added at plating time in the culture medium for 24 h. After being solubilized in 100% DMSO (Sigma-Aldrich, St. Louis, MO), LRRK2 inhibitors were added directly into the culture medium 15 min before IFN-γ treatment. The final concentration of DMSO was of 0.1%. Unless specified in the text, cells were collected 24 h after plating.

### Monocyte maturation towards dendritic cells and macrophage phenotypes

Two millions CD14^+^ CD16^±^ monocytes were plated in 2 mL of culture medium supplemented with 50 µM 2-mercaptoethanol (Sigma-Aldrich, St. Louis, MO), 100 ng/ml (100 IU/ml) recombinant human IL-4 (ImmunoTools, Friesoythe, D) and 100 ng/ml (100 IU/ml) recombinant human GM-CSF (ImmunoTools, Friesoythe, D) for dendritic cells phenotypes and with 20 ng/ml LPS (from *E.coli* O111:B4, Sigma-Aldrich, St. Louis, MO) for macrophage phenotypes. Cells were incubated at 37°C in a humidified atmosphere containing 5% CO_2_. The cultures were fed with fresh complemented culture medium with cytokines or LPS every 2 days. After 7 days, cells were harvested and analyzed by FACS with a direct staining (FITC anti-CD83, PE anti-CD80, PerCP Cy5.5 anti-CD14, APC anti-CD16 and FITC anti-CD103, PE anti-CD206, PerCP-Cy5.5 anti-CD14, APC anti-CD16, FITC anti-CD40, PE anti-CD68, APC anti CD71. For LRRK2 expression analysis by Western blot cells were lysed as described in the following section.

### Western blot analysis

Freshly purified cells were lysed using RIPA buffer complemented with protease inhibitor (Roche Diagnostics, Indianapolis, IN) and phosphatase inhibitor cocktail (Thermo Scientific, Rockford, IL). Samples were sonicated then centrifuged at 13,000 g for 15 min at 4°C. Supernatants were collected and kept at -20°C until use. For *in vitro* experiments, hPBMC or CD14^+^CD16^±^ adherent and non-adherent cells were collected separately at the end of the experiment. Non-adherent cells were removed directly from culture medium by centrifugation and washed once with ice-cold PBS. Adherent cells were collected after being washed twice with cold PBS containing 1 mM of EDTA. Cell pellets were thereafter treated as freshly purified cells. After protein determination using Pierce BCA (Thermo Scientific, Rockford, IL), 25 µg of protein was separated on NUPAGE® Novex 3–8% Tris-Acetate Gel (InVitrogen Ltd, Paisley, UK) or 10–20% Tris-Glycine Gel (InVitrogen Ltd, Paisley, UK) and transferred to nitrocellulose membrane (InVitrogen Ltd, Paisley, UK). Membranes were blocked using 5% non-fat powdered milk (Rapilait, Migros, Switzerland) in PBS buffer containing 0.1% Tween 20 (Merck KGaA, Darmstadt, Germany) for chemiluminescence and with Odyssey® blocking buffer (LI-COR Biosciences, Lincoln, NE) for fluorescence. The membrane was incubated overnight at 4°C with primary antibody. After rinsing in PBS containing 0.1% Tween 20, membranes were incubated with secondary antibody for 2 h at room temperature, processed with an enhanced chemiluminescence detection system (Amersham Biosciences, GE healthcare, little Chalfont, UK) or directly scanned with the infrared Odyssey® imaging system (LI-COR Biosciences, Lincoln, NE). The level of expression of each protein was quantified from scanned images using Odyssey software with average top/bottom background method and fluorescence converted in integrated intensities. The protein signal was normalized with the signal of actin.

### Quantitative reverse-transcription polymerase chain reaction (qRT-PCR)

Freshly isolated cells or cells maintained in culture for 3, 6 or 24 h of induction cells were washed twice with cold PBS and total RNA was prepared with TRIzol Reagent (InVitrogen 15596) using the manufacturer's protocol. 2 µg total RNA was used as template for reverse transcription with qScript cDNA SuperMix 5x (#95048-100 Quanta). cDNA was then diluted 30x and real-time PCR reactions were performed in triplicates using FastStart Universal SYBR Green Master mix (ROCHE #04913914001) and the QuantiTect® primer assays from QIAGEN (Figure S1).

### Statistical analyses

A two-tailed unpaired t-Test was used for comparison between two groups. For experiments with more than two conditions, data were analyzed using one-way analysis of variance (ANOVA) using GraphPad Prism 5. For all analyses, a two tailed test was employed and a Bonferroni' multiple comparison test used as post test. Statistical significance was established at a P<0.05. Data are presented as mean ± standard deviation (SD).

## Results

### LRRK2 expression in hPBMC and hPBMC sub-populations

**Figure 1:**
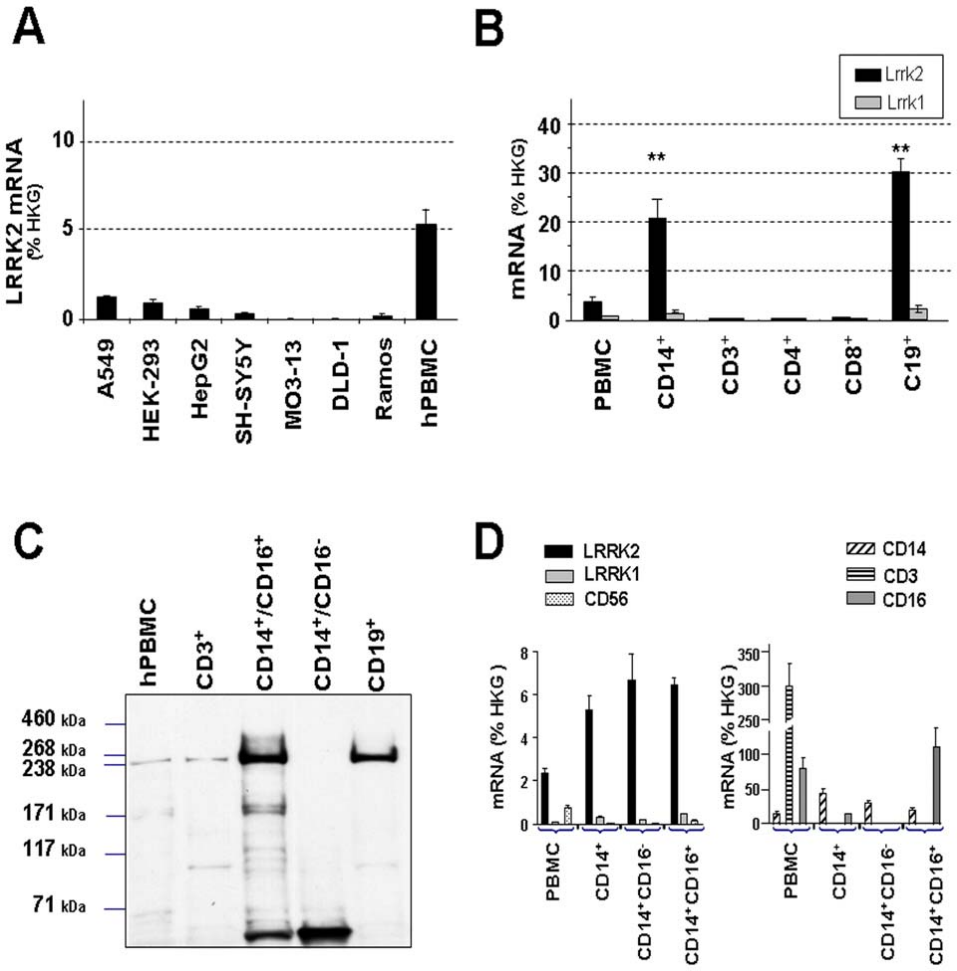
LRRK2 mRNA and protein expression in hPBMC subpopulations. (**A**) LRRK2 mRNA expression in various cell types showing higher expression level in hPBMCs. (**B**) LRRK2 (black bars) and LRRK1 (grey bars) mRNA expression in hPBMC sub-populations. hPBMC sub-population were purified by FACS based on their cell surface markers: Monocytes (CD14^+^), B lymphocytes (CD19^+^) and T-lymphocytes (CD3^+^, CD4^+^ or CD8^+^). Expression level was performed on freshly isolated cells and the graph represents a pool of at least three independent experiments. LRRK2 and LRRK1 mRNA amounts were normalized to average values of three housekeeping genes namely GAPDH, HMBS and actin B (% HKG). LRRK2 mRNA quantities in hPBMC sub-populations were compared to LRRK2 mRNA values in total hPBMC (** p<0.01, n = 3, One-way-Anova with Bonferroni post test). (**C**) Representative western-blot of five independent experiments using ab60937 antibody from (Abcam) confirming LRRK2 protein expression in hPBMC sub-populations with striking enrichment in the CD14^+^CD16^+^ monocyte sub-population as compared to CD14^+^CD16^−^ monocytes. Similar results were obtained with two other antibodies against LRRK2 (Cf. Figure S2). Molecular size markers are indicated on the left in kilo Dalton (kDa). (**D**) LRRK2, LRRK1 and PBMC subtype mRNA expression in purified sub-populations. Graph represents a pool of two independent experiments. Note the identical level of LRRK2 mRNA in CD14^+^CD16^−^ and CD14^+^CD16^+^ monocytes. CD3, CD14, CD16 and C56 mRNA level were used to assess the enrichment efficacy. mRNA amounts were normalized to average values of three housekeeping genes (% HKG).

Recent publications and expression database analyses highlight the fact that LRRK2 mRNA is abundantly expressed in spleen and B lymphocytes [12], [14]. In this study we further evaluated LRRK2 expression in PBMCs at the mRNA and protein levels. We detected a robust LRRK2 mRNA signal in hPBMCs as compared to various cell lines studied (Fig. 1A).

LRRK2 expression in circulating B lymphocytes has recently been described [14]–[15]. To explore if B cells are the only blood cells expressing LRRK2, we studied its expression on isolated PBMC sub-populations (Fig. 1B). PBMC sub-populations were isolated by FACS based on the expression of CD markers and analyzed by qPCR for LRRK2 and LRRK1 expression. First, we confirmed expression of LRRK2 in circulating B cells (CD19^+^). In B cells, LRRK2 was expressed slightly higher in C19**^+^**CD27**^+^** than in CD19**^+^**CD27**^−^** cells (data not shown). In contrast, CD3**^+^**, CD4**^+^** and CD8**^+^** cells representing T cell populations demonstrated very low levels of LRRK2 mRNA. LRRK1 mRNA was found also at very low levels in hPBMCs, and generally in all of the subpopulations investigated. The monocyte population that expressed the LPS co-receptor CD14 revealed high levels of LRRK2 mRNA.

LRRK2 expression was also studied at the protein level (Fig. 1C). The identity of the LRRK2 immunoreactive band was established using three different LRRK2 antibodies (Figure S2). To further document LRRK2 expression in monocytes subpopulations, monocytes were separated accordingly to the low-affinity FcγRIII receptor (CD16) expression (see Figure S3 for FACS profile). In B- or T-lymphocytes LRRK2 protein levels matched LRRK2 mRNA levels, confirming LRRK2 protein expression by B-lymphocytes. For CD14^+^ monocytes we observed a striking difference in LRRK2 protein expression in CD14^+^CD16^+^ as opposed to CD14^+^CD16^-^ monocytes. Both subsets expressed similar levels of LRRK2 mRNA (Figure 1D) but CD14^+^/CD16^+^ cells were found to contain 14.1±6.2 times more (p<0.01, n = 3, Student t-Test) LRRK2 protein than CD14^+^/CD16^−^ cells, where LRRK2 is almost not detectable. This difference between protein levels was confirmed with all three tested antibodies (Figure S2) and was observed whether cells were isolated by FACS (2 independent experiments) or by immunomagnetic beads (3 independent experiments). Furthermore qPCR analysis of these CD14^+^CD16^−^ and CD14^+^CD16^+^ populations confirmed the lack of contamination by CD3^+^, CD19^+^ or CD56^+^ cells (Figure 1D).

### Induction of LRRK2 expression in hPBMC

**Figure 2:**
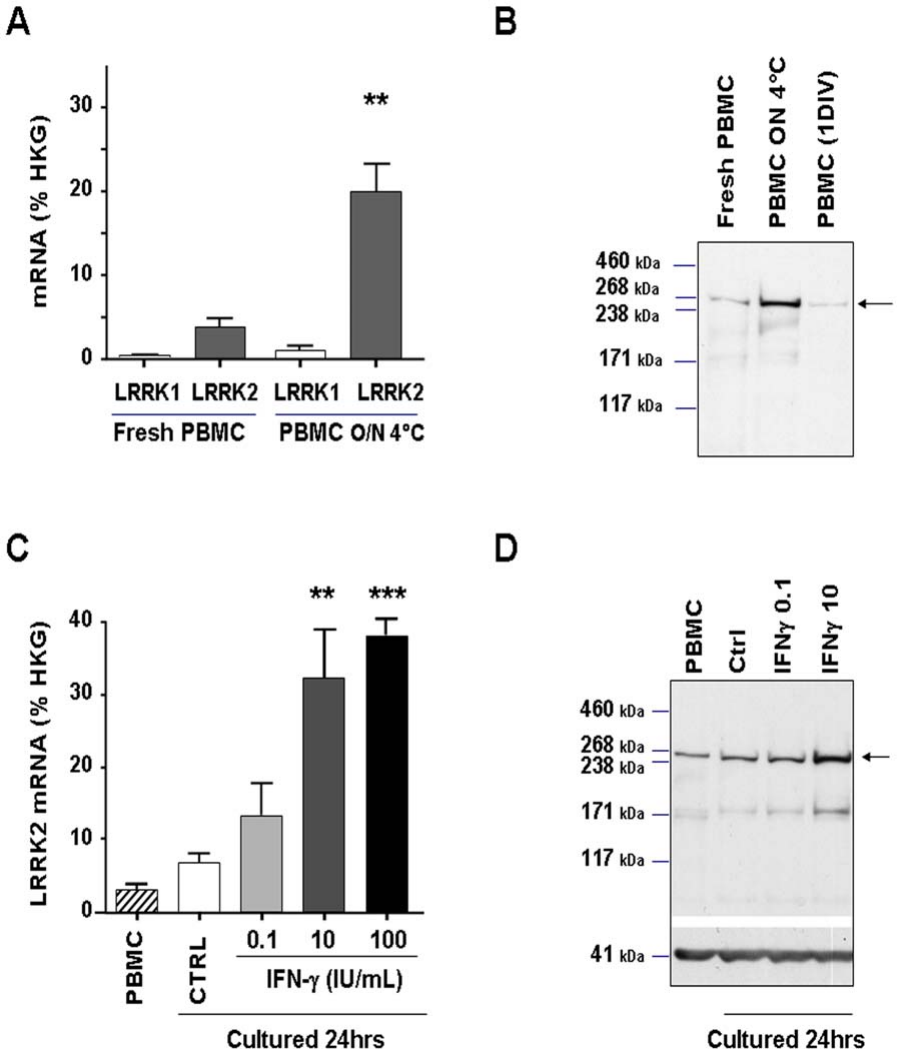
Induction of mRNA and protein LRRK2 expression in hPBMCs. (**A**) LRRK2 and LRRK1 mRNAs from freshly isolated hPBMCs or hPBMCs kept overnight at 4°C were quantified by qPCR (5 independent experiments). (**B**) Western-blot confirming LRRK2 protein induction by hPBMCs overnight storage at 4°C. One day after plating (1 DIV) LRRK2 protein is back to initial value. (**C, D**) IFN-γ induced LRRK2 expression in hPBMC. PBMCs were cultured for 24 h in presence of various amount of IFN-γ (0.1, 10 and 100 IU/mL) and analyzed for LRRK2 mRNA (C, n = 3) and protein (D, n = 4) expression level. PBMC refers to freshly prepared hPBMC and control refers to PBMC cultured for 24hrs in absence of stimulus. Actin protein was used as internal control for equal loading. LRRK2 and LRRK1 mRNA amounts were normalized for housekeeping genes (% HKG). Statistical difference in normalized LRRK2 or LRRK1 mRNA amounts were calculated against values obtained from freshly isolated cells (* p<0.05; ** p<0.01; ***< p0.005, Student t-Test for (A), One-way-Anova with Bonferroni post test for (C).

The proportion of CD14^+^CD16^+^, now referred as nonclassical monocytes is increased during inflammatory conditions and these cells are considered to be more mature than CD14^+^CD16^−^ monocytes [16]–[17]. Since it was previously suggested that LRRK2 belongs to a family of stress-activated kinases [11] we next tested whether LRRK2 protein and mRNA expressions were modulated during induced stress, or during monocyte activation. In order to evaluate expression within a “native" environment that allows potential cross-talk between various PBMC sub-types we first studied LRRK2 induction in hPBMC preparations. We explored a wide range of inducers of cellular stress and cytokines, such as storage at 4°C, H_2_O_2_, LPS, IFN-γ, IFN-β, TNF-α, IL-1β, IL-6, IL-2, IL-15 and IGF-1. Of those tested, only few led to a robust and consistent increase of LRRK2 levels as assessed by qPCR and Western blot (Table 1, Fig. 2 and Figures S4 and S5).

**Table 1:** Effect of various cytokines and stress conditions on LRRK2 expression in hPBMCs.

	Tested molecule			
	IFN-γ (30 IU/mL)	IFN-β (100 IU/mL)	TNF-α (10 ng/mL)	IL-6 (10 ng/mL)
LRRK2 protein	Increase	Increase	Increase	Slight increase
Confirmed	20x/23 expts	2x/2 expts	5x/7 expts	2x/4 expts
LRRK2 mRNA	Increase	Increase	Increase	ND

	Tested molecule			
	IL-1β (25 ng/mL)	IL-15 (10 ng/mL)	IL-2 (10 ng/mL)	IGF-1 (25 ng/mL)
LRRK2 protein	No effect	No effect	No effect	Slight decrease
Confirmed	3x/4 expts	2x/2 expts	1x/1expt	2x/3 expts
LRRK2 mRNA	No effect	ND	ND	No effect

	Tested molecule			
	4°C Overnight	Serum free	H_2_O_2_ (30 µM)	LPS (50 ng/mL)
LRRK2 protein	Increase	Increase	Increase?	Decrease
Confirmed	5x/5 expts	3x/3 expts	2x/5 expts	4x/7 expts
LRRK2 mRNA	Increase	ND	ND	No effect

Freshly prepared hPBMCs were cultured under various conditions for 24 h. LRRK2 protein expression was monitored by Western blot and qPCR. As hPBMC population composition may vary by donor, the number of times where a given result was found as well as the total number of experiments (expts) performed are indicated. Results are only qualitatively represented. Corresponding qPCR data and western blots and are shown in Figures S4 and S5. IFN-γ, IFN-β and to a lesser extent TNF-α were very potent inducers of LRRK2 expression at both the protein and mRNA level. ND: Not determined.

In the stress inducing category, we found that keeping PBMCs overnight at 4°C in PBS solution led to a robust >5-fold increase (p<0.01, n = 5) of LRRK2 mRNA (Fig. 2A) and protein (Fig. 2B) as compared to freshly prepared hPBMCs. Interestingly, 24 h after plating LRRK2 levels were back to the level found in freshly isolated cells (Fig. 2B).

Since cytokines and interferons are major players in monocyte activation we next tested their potential to increase LRRK2 expression in hPBMCs. IFN-γ treatment increased LRRK2 mRNA and protein expression respectively by 11.4±3.5 fold (p<0.005, n = 6) and 6.2±2.1 fold (p<0.005, n = 5; Fig. 2C, 2D). This was dose- (Fig. 2C) and time dependent with a peak of mRNA expression at 6 h (n = 3, Figure S4). Other molecules such as IFN-β, TNF-α or IL-6 also induced LRRK2 albeit only to a modest degree and with donor-to-donor variation (Table 1 and Figure S5). In contrast, treatment of hPBMC with LPS, IL-1β or IGF-1 did not induce LRRK2. Of note, none of the treatments described above significantly induced LRRK1 mRNA expression (Figure S5).

**Figure 3:**
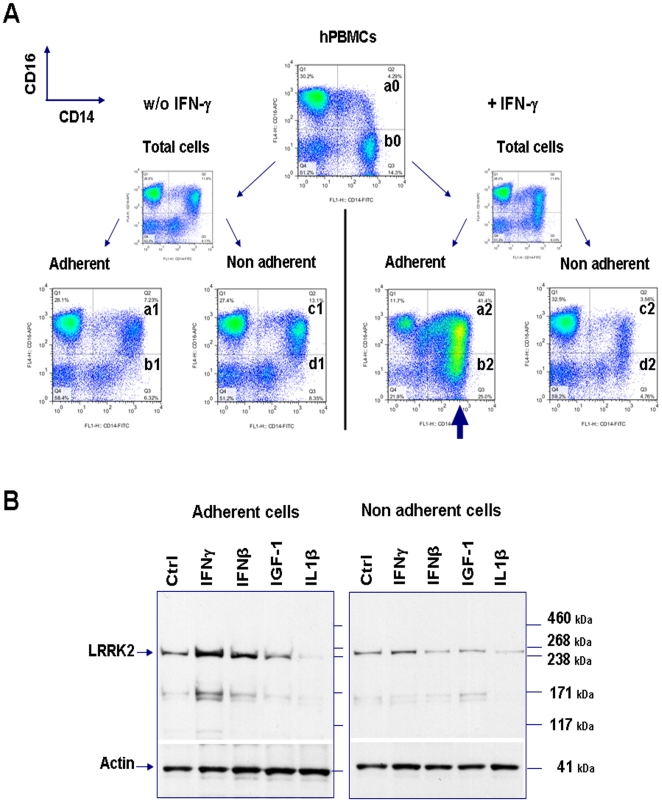
IFN-γ induced expression of LRRK2protein in CD14^+^ monocytes. Twenty four hours after plating with or without cytokines, adherent and non-adherent hPBMCs were separately collected and processed for FACS analysis for CD14 and CD16 populations (**A**) and Western-blot analysis for LRRK2 protein level (**B**). (**A**) Percentage of CD14^+^ adherent cells was calculated as the ratio of panel (a+b)/((a+b)+(c+d)) for each condition. Percentage of CD16^+^/CD14^+^ adherent cells was calculated as the ratio of a/(a+b) for each condition. The arrow highlights the very strong shift of cells toward a CD14^+^ CD16^+^ population after IFN-γ treatment (30 IU/mL). (**B**) Effect of IFN-γ (30 IU/mL) or IFN-β (100 IU/mL) on LRRK2 expression is restricted to adherent cells population (mainly constituted of CD14^+^ cells). By contrast, IGF-1 (25 ng/mL) or IL-1β (25 ng/mL) had no effect on LRRK2 expression. Actin protein was used as loading control. Representatives western blot of three independent experiments.

It has been reported previously that IFN-γ treatment provokes adhesiveness of monocytes and a switch in the CD14^+^CD16^+^/CD14^+^CD16^−^ ratio whereas non-activated monocytes, NK, B and T cells remain in suspension [18]. We used this phenomenon to further explore which cells induce LRRK2 expression after IFN-γ stimulation. In our experiments at plating time the monocyte (CD14^+^) population represented 15–20% of the total plated cells. At this moment only a quarter of these monocytes were CD16^+^. Twenty four hours after IFN-γ treatment, 60-80% of adherent cells were monocytes and two-thirds of them CD16^+^ (Fig. 3A). In adherent cells the increase of LRRK2 protein levels was very significant (Fig. 3B) and paralleled the adhesion of almost 90% of CD14^+^ cells (Fig. 3A). These adherent cells corresponded to the population of activated monocytes as assessed by high ICAM-1 (CD54), low L-selectin (CD62L) and a shift in co-stimulatory ligand for T cells activation (CD80) co-expression with CD14 and CD16 markers (Figure S6). Therefore the IFN-γ -induced LRRK2 protein expression paralleled monocytes adhesion and switch towards CD16 expression. In contrast to IFN-γ, IGF-1 and IL-1β did not induce LRRK2 expression in adherent or non-adherent populations (Fig. 3B).

### LRRK2 expression by maturating monocytes, macrophages and dendritic cells

**Figure 4:**
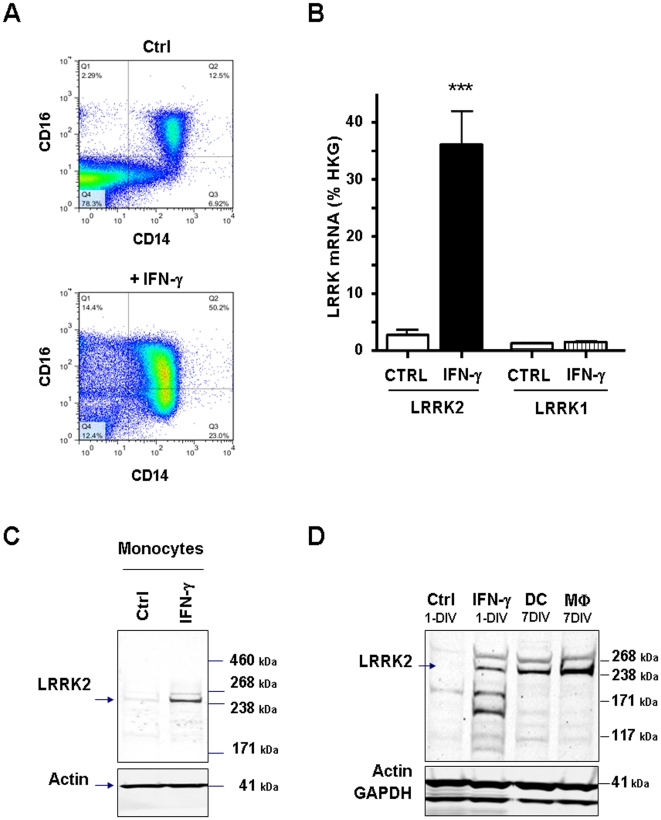
LRRK2 expression by maturating monocytes, macrophages and dendritic cells. (**A**) Purified CD14^+^ monocytes were treated with IFN-γ (30 IU/mL) for 24 h and cells analyzed by FACS to assess CD14 and CD16 expression levels. (**B**) and (**C**) The observed shift towards CD14^+^CD16^+^ cells observed in (A) paralleled an increase in LRRK2 mRNA and protein levels as revealed by qPCR (n = 3) and Western blot (n = 4) (***, p<0.005, Student t-Test). (**D**) LRRK2 expression persists in monocytes-derived dendritic cells (DC) and macrophages (MФ) matured 7 days *in vitro* with IL-4/GM-CSF (dendritic cells) or LPS (macrophages). Cell type was assessed by FACS analysis of CD markers (Cf. Figures S8 and S9). Representative western blot of at least 3 independent experiments are shown.

To establish if the induction of LRRK2 expression by CD14^+^CD16^+^ cells in response to IFN-γ was due to a direct or indirect effect of IFN-γ on CD14^+^ cells, we purified CD14^+^ populations and treated them with IFN-γ. We found that on purified monocytes the IFN-γ-induced shift of CD14^+^CD16^-^ to CD14^+^CD16^+^ monocytes (Fig. 4A) was indeed accompanied by an increase in LRRK2 mRNA and protein expression (Fig. 4B, C) by respectively 13±4 (p<0.005, n = 3) and 17.5±9.8 (p<0.01, n = 4) fold. Another condition known to directly activate monocytes is to plate cells in serum-free media. In these conditions monocyte activation was also associated with a significant increase of LRRK2 protein expression in adherent cells (Figure S7).

These experiments suggest that LRRK2 mRNA and protein are expressed during monocyte activation. As blood monocytes represent an intermediate population that differentiates into either macrophages or dendritic cells [19]–[20], we also studied LRRK2 expression during *in vitro* maturation of monocytes towards dendritic cells and macrophages. The purified CD14^+^ monocyte population was cultured for one week in presence of GM-CSF/IL-4 or LPS to generate dendritic cells and macrophages respectively. Monocyte differentiation was assessed by FACS determination of macrophage and dendritic cells markers (Figures S8 and S9) such as CD40, a costimulatory protein found on antigen presenting cells CD68, a glycoprotein binding to low density lipoprotein, transferrin receptor protein 1 (TfR1) also known as CD71, T-activation co-stimulatory factor (CD80), Sialic acid binding Ig like adhesion receptor (CD83), Integrin αE (CD103) and Macrophage Mannose Receptor (CD206). High levels of LRRK2 protein were detected in both dendritic cells and macrophages (Fig. 4D) suggesting that LRRK2 protein levels remains elevated during the maturation process.

Altogether these data revealed the modulation of LRRK2 mRNA and protein levels by IFN-γ and the preferential expression of LRRK2 protein by CD14^+^CD16^+^ monocytes and by matured DCs and macrophages.

### LRRK2 inhibition decreases CD14, CD16 and MHC-II expression by monocytes

Because the IFN-γ -induced LRRK2 increase in monocytes paralleled the switch toward CD16^+^ cells, we explored the effect of kinase inhibitors active on LRRK2. The most selective inhibitor described to date is IN-1 with an IC_50_ of 13 nM on LRRK2 wt kinase activity inducing a complete dephosphorylation of LRRK2 Ser910/Ser935 at 1 and 3 µM [21]. We also tested inhibitors from different chemical families (Table 2), which all have in common to inhibit LRRK2 kinase activity, but differ in their kinase selectivity profile and potency towards LRRK2 kinase inhibition.

**Table 2:** Effect of LRRK2 inhibitors on IFN-γ-induced expression of CD14, CD16 and MHC-II by monocytes.

			%CD14+ cells		%CD16+ cells		%MHC-II+ cells		
CTRL +	**DMSO 0.1%**	**ND**	**31±12**		**22±12**		**31±20**		n = 8
INF-γ +	**DMSO 0.1%**	**ND**	**80±5^c^**		**65±11^c^**		**75±8^c^**		n = 8

		Enzyme	%CD14+ cells @		%CD16+ cells @		%MHC-II+ cells @		
		IC50 (nM)	1 µM	10 µM	1 µM	10 µM	1 µM	10 µM	
INF-γ +	**K252a**	4	**50±10** ^a^	**0.4±0.3** ^c^	**45±8**	**1±1** ^c^	**31±11** ^c^	**19±5** ^c^	n = 3
INF-γ +	**1N-1**	13	**76±2**	**32±7** ^c^	**10±3** ^c^	**3±1** ^c^	**56±5**	**47±10** ^c^	n = 4
INF-γ +	**Sunitinib**	79	**79±6**	**9±6** ^c^	**79±9**	**7±5** ^c^	**53±14**	**18±7** ^c^	n = 4
INF-γ +	**H-1152**	244	**72±14**	**76±13**	**63±15**	**63±13**	**66±15**	**68±15**	n = 5
INF-γ +	**Y-27632**	2300	**67±18**	**67±17**	**61±23**	**61±21**	**54±10**	**59±16**	n = 3

For each inhibitor IC_50_ obtained on LRRK2 WT kinase enzyme activity is indicated (Deng et al., 2011; Nichols et al., 2010). Is indicated the percentage +/− SD of CD14^+^, CD16^+^ or MHC-II^high^ cells obtained after IFN-γ treatment (30 IU/mL) in presence of 1 or 10 µM of LRRK2 inhibitors. In all experiment DMSO concentration has been fixed to 0.1%. (**^a^** p<0.05, **^c^** p<0.005, One way Anova with Bonferroni post test). N indicates the number of independent experiment for each tested compound. ND: Not determined.

**Figure 5:**
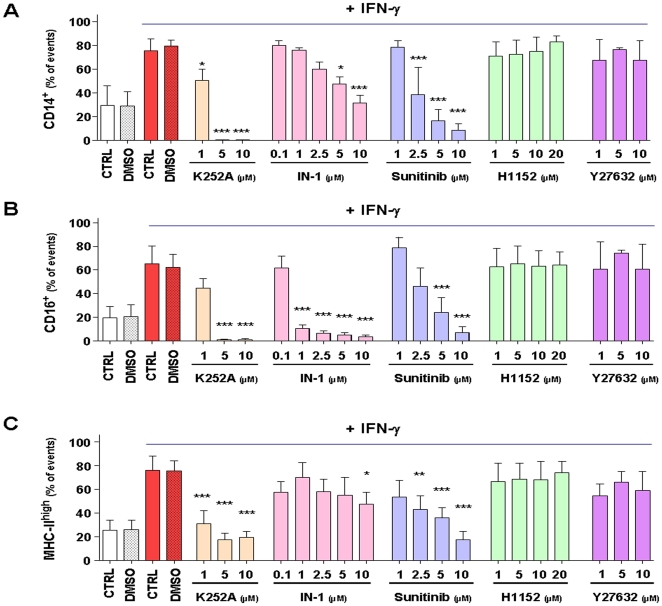
LRRK2 inhibition decreased IFN-γ–induced CD14, CD16 and MHC-II expression by monocytes. Purified monocytes were cultured in presence of various concentrations of LRRK2 inhibitors before stimulation with IFN-γ. Twenty four hours later cells were collected and analyzed by FACS for quantification of CD14 **(A)**, CD16 **(B)** and MHC-II **(C)** expression levels. For details on FACS settings see Figure S10. Values are expressed as the number of cells with a given phenotype (e.g. CD14^+^, CD16^+^ or MHC-II^high^) versus the total number of cells analyzed. Data of several experiments have been pooled and at least three independent experiments have been performed for each dose tested (for more details cf. Figure S10 and Table-2). Most potent LRRK2 inhibitors (K252A, IN-1 and Sunitinib) induced a severe reduction of CD14 (A) and CD16 (B) expression by monocytes meanwhile less potent inhibitors (H1152 and Y27632) had no effect. Inhibition of MHC-II expression (C) was also observed with best LRRK2 inhibitors. (* p<0.05, ** p<0.01, ***p<0.005, One-way-Anova with Bonferroni post-test).

Twenty four hours after plating in presence of LRRK2 inhibitors and IFN-γ cells were collected and stained for CD14, CD16 and MHC-II expression and analyzed by FACS (Figure S10). IN-1, the most selective inhibitors tested [21], [22], dose-dependently blocked the CD14^+^ (Fig. 5A) and CD16^+^ (Fig. 5B) switch in response to IFN-γ treatment (Table 2). Effects of IN-1 on CD16 expression were still very significant at 1 µM (p<0.005), a concentration at which IN-1 is believed to be quite selective. These effects on CD14^+^ and CD16^+^ switch were confirmed with two other very potent LRRK2 inhibitors namely K252a and Sunitinib that have an IC_50_ of respectively 4 nM and 79 nM [22]. Indeed, at 10 µM not only IFN-γ effects were fully blocked (p<0.005 for all three inhibitors) but also the percentage of CD16^+^ cells dropped below untreated values (p<0.01 for all three inhibitors). At 10 µM, MHC-II expression was also attenuated by these inhibitors (p<0.01 for IN-1, p<0.005 for K252a and Sunitinib; Fig. 5C, Table 2). In contrast, H1152 (IC_50_: 244 nM) and Y27632 (IC_50_: 2,300 nM) two inhibitors with 20-100 -fold lower potency on LRRK2 than IN-1 had no effect on IFN-γ-induced monocyte maturation (Fig. 5A, B, C and Table 2).

**Figure 6:**
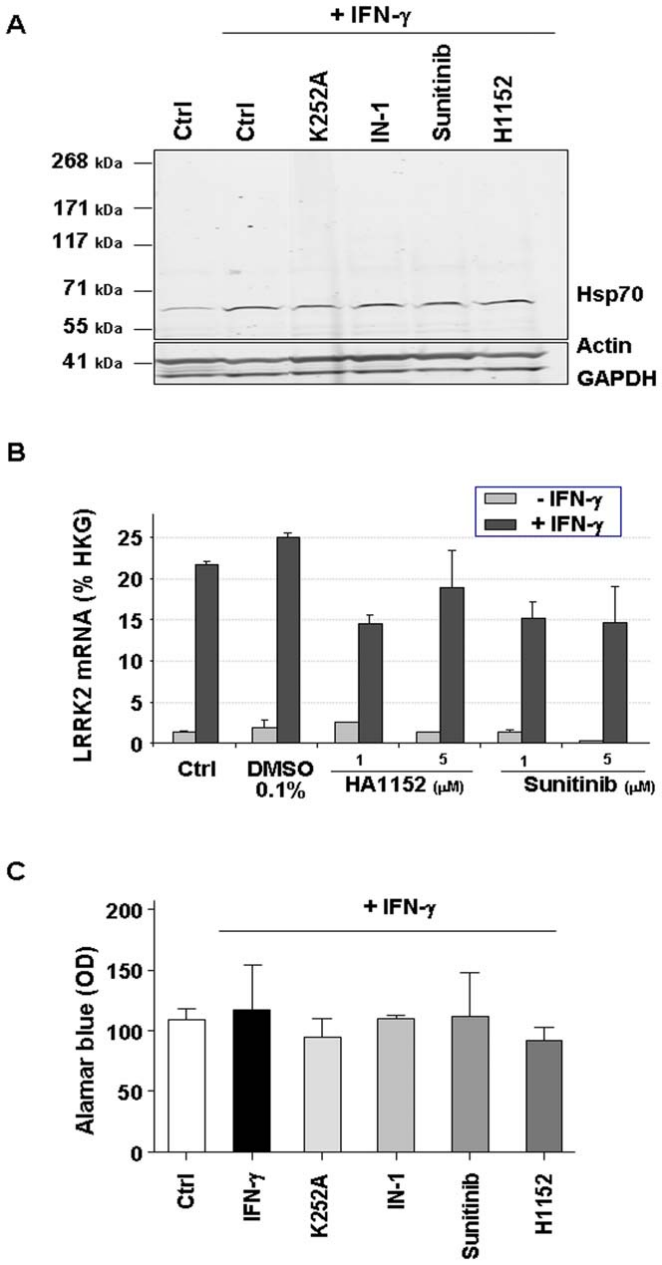
LRRK2 inhibitors were not shunting IFN-γ induction. hPBMC-derived monocytes were cultured for 24 h in presence of LRRK2 inhibitors plus IFN-γ (30 IU/mL). Effect of IFN-γ on monocytes was assessed by monitoring the induction of Heat-shock protein Hsp70 by western blot **(A)** and by quantifying LRRK2 mRNA by qPCR **(B)**. LRRK2 inhibitors did not prevent Hsp70 or LRRK2 induction. **(C)** Effect of LRRK2 inhibitors (10 µM for each) on cell survival was monitored by Alamar Blue 24 h after plating. Graphs represent an average of three independent experiments. No statistical difference was found between control (ctrl) and other groups (One-way-Anova, Bonferroni post test).

In order to ascertain that the used inhibitors were not just shunting IFN-γ responses we looked at several proteins known to be induced by IFN-γ as well as at LRRK2 mRNA induction by IFN-γ. None of the tested inhibitor blocked IFN-γ-induced Hsp70 expression by monocytes (Fig. 6A) nor prevented LRRK2 mRNA induction in hPBMC (Fig. 6B). The compounds also did not affect cell viability as assessed by Alamar Blue tests at 24 h (Fig. 6C).

## Discussion

Autosomal-dominant mutations in LRRK2 alias Dardarin significantly raise the risk of familial PD, and the G2019S mutation is also found frequently in sporadic PD patients. The functional consequences of these mutations remain largely unknown and most of the available data have been generated using artificial systems based on ectopic expression of wild-type LRRK2, or LRRK2 mutations associated with PD.

In the present study, we explored LRRK2 expression in hPBMC. By sub-fractionating PBMCs two cell populations expressing LRRK2 protein were identified namely B cells (CD19**^+^**) and a sub-set of monocytes (CD14**^+^**CD16**^+^**). In contrast, T cell (CD3^+^, CD4**^+^**, CD8**^+^**) and NK cell (CD3**^-^**CD14**^-^**CD16**^+^**) populations did not express LRRK2. These results are partially in agreement with recent data published [14], where a strong expression of LRRK2 in CD19**^+^** splenic cells and a lack of expression in CD3**^+^** cells were described. Interestingly, in this study a weak expression in CD11b**^+^** splenic cells (a CD marker for macrophages but also other cell types e.g. neutrophils and NK cells) was described, whereas we found a robust expression of LRRK2 protein in CD14**^+^**CD16**^+^** circulating cells but not in CD14**^+^**CD16**^−^** cells.

We did not found differences between LRRK2 mRNA levels in CD14**^+^** CD16**^−^** and CD14**^+^** CD16**^+^** subsets. We further document our finding by a search on the transcriptome analysis of CD14**^+^** CD16**^-^** versus CD14**^+^** CD16**^+^** cells by Ingersoll et al. [23]. Their transcriptome data confirmed that LRRK2 mRNA level is similar in these two sub-populations (Figure S11). As we found a differential LRRK2 protein expression using three distinct antibodies, we believe that this difference between LRRK2 mRNA and LRRK2 protein is real. Whether it reflects a lack of translation of LRRK2 mRNA or a lack of LRRK2 protein stabilization in CD14**^+^** CD16**^−^** cells remains to be elucidated. In contrast to what we have seen in CD14**^+^** CD16**^−^** monocytes, in B-lymphocytes high LRRK2 mRNA level was reflected by a high LRRK2 protein level.

There are further subsets of CD14^+^ monocytes that are distinguished by their CD16 expression levels. There is a developmental relationship between these three subsets, as classical (CD14**^+^** CD16**^−^**) matured towards noncalssical (CD14^+^/CD16^++^) via an intermediate stage (CD14**^++^**CD16**^+^**) [17]. Nonclassical monocytes represent less than 10% of the monocyte population in hPBMCs and are perceived as crucial in inflammation and infectious diseases in man. Indeed CD14**^+^**CD16**^++^** monocytes have higher levels of HLA-DR [24] and display higher antigen presenting cell (APC) activity than CD14**^+^**CD16**^−^** cells (for review see [25], [16]). Several publications showed that CD14**^+^**CD16**^++^** preferentially develop into dendritic cells [26] and produce higher level of TNF-α and IL-1 than CD16^-^ monocytes [27]–[28]. In the current study we did not separate the two subsets of CD16^+^ monocytes (CD14**^++^**CD16**^+^** versus CD14**^+^**CD16**^++^**) and therefore will only talk about CD14**^+^**CD16**^+^** and CD14**^+^**CD16**^−^** monocytes subpopulations. In future studies to resolve the role of LRRK2 it might be worth to distinguish these two subsets.

The difference we observed between LRRK2 levels in monocyte sub-types suggests that LRRK2 expression is regulated during the monocytes/macrophages life cycle. The finding that plating PBMCs in serum-free media (a classical paradigm to activate monocytes) induced LRRK2 expression strengthens this idea. Expression of LRRK2 by dendritic cells and macrophages in Crohn's disease patients has recently been reported [29]. Our *in vitro* maturation experiments confirm the observation that maturation of monocytes towards DC or macrophages is accompanied by LRRK2 expression. Finally, recent data showing that pre-B, B1 and B2 subtypes of B-lymphocytes express different levels of LRRK2, indicate that LRRK2 expression is also regulated during B cell maturation [15]. Overall, ours as well as others' data point to a role of LRRK2 in cell differentiation and cell fate of B lymphocytes and monocytes lineage.

The second key point of this study is the demonstration that IFN-γ induces of LRRK2 expression in hPBMC, and more specifically in isolated monocytes. Gardet *et al.*, [29] also reported that IFN-γ could induce LRRK2 expression on hPBMC and THP-1 cells. In the current study we confirmed these finding and demonstrate that IFN-γ-induced increase of LRRK2 levels in hPBMCs was attributable to adherent CD14**^+^** cells since in non-adherent cells mostly composed of CD4**^+^**, CD8**^+^**, CD19**^+^** and CD3**^-^**CD14**^-^**CD16**^+^** cells no increase of LRRK2 was detected. The similarity between this study and ours, especially regarding the amplitude of the induction, is striking and strongly confirms control of LRRK2 expression by IFN-γ. However, we did not detect LRRK2 expression in T lymphocytes whether or not they were treated with IFN-γ. This lack of LRRK2 expression by T lymphocytes is in accordance with Maekawa's observations [14].

IFN-γ signaling is a gate-keeper of the innate immune system and its ability to activate monocytes is well documented. For example, IFN-γ induces monocytes to express several markers of maturation such as I-CAM, MHC-II, CD14, CD16 [18], [30]. In our experiments LRRK2 induction paralleled CD14**^+^** and CD16**^+^** shift and MHC-II expression by monocytes. With respect to LRRK2, IFN-γ seems to have two different effects. First it induces LRRK2 mRNA expression within few hours. This effect could be due to a direct effect of IFN-γ on LRRK2 transcription, and in line with this notion we and others [29] found that the 5′ region of the LRRK2 gene contains several consensus interferon-stimulated responsive elements (IRSE), and gamma activated sequences (GAS) (Figures S12 and S13, [31]). It is interesting to note that IRES and GAS sequences were not found in the first 2 kbp of the LRRK1 gene promoter (Figure S14). Second, IFN-γ is known to induce HSP90 expression [32] and HSP90 might stabilize LRRK2 posttranslationally by preventing LRRK2 from CHIP mediated proteasomal degradation as previously suggested [33], [34], [35]. Our experiments with Geldanamycin confirmed that LRRK2 stabilization is HSP90 dependent and that IFN-γ induction of LRRK2 protein requires HSP90 (data not shown). It should be noted that IFN-γ is not the only inducer of LRRK2, indeed in our experiments other stress conditions such as serum free culture or addition of cytokines such as IFN-β or TNF-α, also induced LRRK2 but in a more variable and weaker manner than IFN-γ.

The third interesting finding in our current study is that potent kinase inhibitors active on LRRK2 such as IN-1, K252a and Sunitinib significantly attenuate the CD14, CD16 and MHC-II shift in expression levels following IFN-γ stimulation. These effects are not due to a shunting of global IFN-γ response since an increase of Hsp70 expression and an induction of LRRK2 mRNA were still observed in presence of these inhibitors. Two other inhibitors, H1152 and Y27632, with much lower potency on LRRK2 inhibition (20–50 times lower [21], [22]) did not significantly prevent monocytes differentiation. Such a difference between LRRK2 inhibitors efficacy has also been described in cellular assays assessing LRRK2 Ser910 phosphorylation [22], [36]. Markedly, these publications also highlighted the fact that despite their nanomolar activity on recombinant enzyme these inhibitors were only active at low micromolar concentrations on cells. Even with the most selective LRRK2 inhibitor IN-1, in cellular assays complete dephosphorylation of LRRK2 ser910/ser935 was observed at 1-3 µM. In this assay, the specificity of IN-1 was assessed by using a drug resistant mutant LRRK2 (A2016T) [21]. There is no explanation for this significant shift in potency but it might reflect compounds capability to enter the cells as well as more complex considerations such as stability and metabolism. With respect to selectivity the recently characterized inhibitor IN-1 appears much more selective while as potent as K252a and Sunitinib [21] and represents therefore an important novel tool to study LRRK2. Indeed it has been evaluated against a panel of more than 470 kinases and to date DCLK2 is the only kinase with an IC_50_ close to those of LRRK2. As in our assay IN-1 is as active at 1 µM than at 10 µM in blocking CD16 expression, we are in a same range of efficacy than what has been found in LRRK2 Ser910/Ser935 cellular assays and believe that our data most probably reflect LRRK2 inhibition. Nevertheless, we keep in mind that none of the other tested inhibitors is exclusively LRRK2 specific and the identification of more selective LRRK2 inhibitors and the use of genetic approach are needed to confirm and extend our findings.

The hypothesis that LRRK2 might be involved in monocyte maturation echoes recent observations made by others. For example, LRRK2 KO mice have a tendency to develop splenomegaly [37]. This might reflect difficulties for immune cells to mature and egress from spleen. It was also reported that B-lymphoblastoid cell lines carrying LRRK2 mutations display impaired growth [38] and that LRRK2 might be associated with increased susceptibility to Crohn's disease [39]. Transcriptional profiling of PBMCs from G2019S LRRK2 PD patients revealed a deregulation in leukocyte extravasation signaling and other immune functions, including an up-regulation of IFN-γ mRNA [40].

The expression pattern of LRRK2 protein and its possible role in immune cell maturation raise the question as to whether LRRK2 constitutes a link between the immune system and Parkinson's disease. Indeed, there is increasing evidence of immune components playing a role in Parkinson's disease progression. Invasion of T cells in the brain and modification of the CD8^+^CD4^+^CD25^+^ ratio have been reported in PD patients [41]–[42]. Several inflammatory cytokines such as IL-2, IL-4, IL-6, IL-10, TNF-α and IFN-γ are up-regulated in sera of PD patients [43]-[44] and association between systemic markers of inflammation and idiopathic PD risk has been reported [45]. Interestingly, increased presence of CD14^+^CD16^+^ cells is commonly associated with inflammatory diseases such as Atherosclerosis, Kawasaki disease or bacterial infection (for review see [16]) and LRRK2 participates to antibacterial responses [29]. In HIV patients with encephalopathy, perivascular macrophages in the brain were found to be CD14^+^CD16^+^ [46]. To our best knowledge there is no study specifically assessing the role of CD14^+^CD16^+^ cells in PD patients, but as these cells can switch to dendritic cells, they might participate in initiation or enhancement of immune responses by activating T cells. The recently discovered genetic association between the HLA region with late-onset sporadic Parkinson's disease [47] strengthens the possible relevance of antigen presenting cells in PD. Interestingly, Gottfried-Blackmore *et al.*, [48] recently described a sub-population of microglia named brain dendritic cells that specifically respond to intracerebral injected IFN-γ by increasing MHC-II expression and induce CD4 T cells to proliferate. Based on our findings, it will be worthwhile to study the role of LRRK2 and its mutations in this context.

In conclusion, our study revealed a preferential expression of LRRK2 protein but not mRNA in activated CD14^+^CD16^+^ monocytes. Monocytes activation by IFN-γ was accompanied by up-regulation of LRRK2 mRNA and protein levels in monocytes. Using potent inhibitors of LRRK2 kinase activity, we also provided evidence that LRRK2 signaling might be involved in expression of cell surface markers associated with monocyte maturation. Our data support previous observations on the possible role of LRRK2 during immune cells maturation and open the possibility to study LRRK2 kinase function in a "natural" biological system. In addition our data might offer a functional pharmacodynamics biomarker for LRRK2 inhibitors activity that could complement measurement of Ser^910^/Ser^935^ phosphorylation of LRRK2 as proposed by Dzamko *et al.* [36].

## Supporting Information

Figure S1 **Reference of qPCR primers used in this study.** Primers were obtained from QIAGEN. Glyceraldehyde 3-phosphate dehydrogenase (GAPDH), hydroxymethylbilane synthase (HMBS) and Actin B were used as internal control (house keeping genes). Leucine-rich repeat kinase 2 (LRRK2), Leucine-rich repeat kinase 1 (LRRK1), cluster of differentiation -3 (CD3), -4 (CD4), -8a (CD8a), -14 (CD14), -16 (CD16) and -19 (CD19). In all following experiments values of other genes were expressed as percentage of these three housekeeping genes.
(TIF)

Figure S2 **Validation of data obtained with the LRRK2 antibody ab60937 from Abcam using two additional LRRK2 antibodies.** Experiments performed with AT106 from Alexis Biochemicals (panel A) or with MJFF3-c69-6 from Epitomics Inc. (panel B), confirmed the higher LRRK2 protein content in CD14^+^CD16^+^ monocytes as compare to CD14^+^CD16^−^ cells. Western blots were performed the same day from the same material. Monocytes sub-populations were purified from hPBMC using immunomagnetic beads and all fractions including starting material (hPBMC) were loaded on the gel. CD14^+^ and CD14^-^ respectively refer to monocytes and other cell types that are not expressing CD14. LRRK2 immunoreactive band is highlighted by the arrow. LRRK2 protein quantification was performed using the LI-COR Odyssey® and results are expressed in function of actin. Note that AT106 is also giving an additional band above LRRK2. As this band was not found using ab60937 or MJFF3-c69-6 antibodies, we did not consider it for LRRK2 quantification.
(TIF)

Figure S3 **FACS assessment of CD14^+^ and CD14** ^+^ **CD16^+^ enrichment from hPBMCs.** FACS sorting of hPBMC sub-populations showing consecutives enrichment in CD14^+^ and CD14^+^/CD16^+^ monocytes. (1) At the beginning total PBMCs contained vast majority of CD14^−^/low cells corresponding to T-lymphocytes (bottom left quadrant) and NK cells (top left quadrant). (2) CD14^+^ enrichment using negative selection discarded most of T and NK cells, leaving a CD14^+^CD16^+^ and CD14^+^/CD16^−^ mixed population. (3) This mixed population could be further worked on to purify CD14^+^CD16^−^ sub-population to homogeneity or to enrich CD14^+^CD16^+^ cells using CD16^+^ positive selection.
(TIF)

Figure S4 **Time-course expression of LRRK2 by hPBMC stimulated by IFN-γ.** At different times with or without IFN-γ (30 IU/mL) treatment cells were collected and LRRK2 mRNA expression quantified by qPCR. Increased expression was compared to time matching control without IFN-γ. (* p<0.05, ** p<0.01, *** p<0.005, Student t-Test, n = 3 for each point).
(TIF)

Figure S5 **Effect of various cytokines on LRRK2 expression by hPBMCs.** (A) and (B) Effect of various cytokines on LRRK2 and LRRK1 mRNA expression by hPBMC was monitored after 24 h of culture. LRRK2 protein content was also assessed (C). IFN-γ (0.1, 10 and 100 IU/mL); LPS (50 ng/mL), TNF-α (10 ng/mL), IGF-1 (25 ng/mL), IL-1β (25 ng/mL), IFN-β (100 IU/mL), IL-2 (10 ng/mL), IL-6 (10 ng/mL), IL-15 10 ng/mL and H_2_O_2_ (30 µM) were added at plating time. PBMC refers to freshly purified PBMC (starting material) and control refers to untreated hPBMC after one day in vitro. Western blot are representative of at least two independent experiments (except for IL-2, Cf. Table-I). mRNA values are expressed as percent of house keeping genes (%HKG). Statistical difference in normalized LRRK2 or LRRK1 mRNA amounts were calculated against values obtained on freshly isolated cells (* p<0.05; ** p<0.01, *** p<0.005, One Way Anova, with Bonferroni post-test; n = 3 for each point).
(TIF)

Figure S6 **Assessment of monocytes activation by IFN-γ treatment of hPBMCs.** Twenty four hours after *in vitro* treatment with IFN-γ (30 IU/mL), total PBMC (top panel) or purified monocytes (bottom panel) were harvested and analysed by FACS for various activation markers. Graph represents the number of cells (Events count) in function of staining intensity (Fluorescence Intensity). The shift towards high level of I-CAM (CD54), a molecule involved in cell adhesion, is characteristic of monocyte activation in response to IFN-γ. Note the similarity between the shift in PBMC and in purified monocytes populations. As lymphocytes also express CD54 another cell adhesion molecule L-selectin (CD62L) was measured. The absence of CD62L shift in response to IFN-γ and the low expression level of CD62L by monocytes confirmed that CD54 increase was due to monocytes activation. Finally, we studied the expression of a co-stimulatory signal ligand for T cells (CD80) expressed on B-cells and monocytes once activated. In PBMC as on purified monocytes we detected a slight shift in CD80 expression. CD80 expression will further increase with former maturation of monocytes.
(TIF)

Figure S7 **Serum free conditions induced LRRK2 expression by hPBMCs.** PBMCs were plated in presence or absence of 10% foetal calf serum. Ninety minutes later medium was complemented with 10% FCS. Cultures were thereafter treated or not with IFN-γ (30 IU/mL) and cells collected 24 h later. LRRK2 protein levels were determined by western blot. Transient plating in serum free conditions induced LRRK2 expression but did not blunt IFN-γ response. Representative western blot of three independent experiments is shown.
(TIF)

Figure S8 **FACS analysis of monocytes maturation towards DC and macrophages.** Purified monocytes CD14^+^CD16^+/−^ populations were matured for 7 days *in vitro* with IL-4/GM-CSF towards dendritic cells (DC) or with LPS towards macrophages (MФ) phenotypes. DC and MФ are very heterogeneous in term of cell surface markers expression. As expected after 7 days of *in vitro* maturation, LPS treated monocytes have a MФ like pattern (brown line) with CD14^High^, CD16^+^, CD80^+^, CD83^+^, CD103^+^ and CD206^High^ meanwhile IL-4/GM-CSF treated monocytes are DC like (black line) with a CD14^Low^, CD16^Low^, CD80^High^, CD83^+^, CD103^+^ and CD206^+^ pattern. The initial pattern obtained with purified monocytes one day after plating (red line) is included to highlight the switch in maturation markers expression.
(TIF)

Figure S9 **FACS analysis of monocytes maturation towards macrophages.** To further document the induction of macrophage phenotype by LPS, additional macrophage markers, namely CD40, CD68 and CD71 were tested. After 7 days *in vitro* with LPS, monocytes CD14^+^CD16^+/−^ populations matured towards macrophages (MФ) phenotypes with elevated expression of CD14^ High^, CD40^+^, CD68^+^ and CD71^+^ (orange line). The initial pattern obtained with purified monocytes one day after plating (red line) is included to highlight the switch in maturation markers expression.
(TIF)

Figure S10 **Effect of LRRK2 inhibitors on IFN-γ-induced cell surface markers by monocytes.** After background determination using non-labeled cells and cells labeled with control isotypes, the threshold was set-up on IFN-γ (B) versus untreated (A) cells. With an average fluorescence value of 1.10^+2^, IFN-γ treatment (light blue line) induced a very strong shift in CD14^+^ (C) and CD16^+^ (D) expression as compare to untreated monocytes (red line). Due to endogenous high expression, MHC-II shift in response to IFN-γ was less obvious but still significant (E). Effects of 10 µM (dark line) and 0.1 µM (light grey) of IN-1 on CD14, CD16 and MHC-II expression were presented. At 10 µM IN-1 blocked IFN-γ -induced CD14, CD16 and MHC-II expression (p<0.005, p<0.005 and p<0.05, respectively. One-way-ANOVA, Bonferroni post-hoc test, n = 3 for each).
(TIF)

Figure S11 **Analysis of transcriptome data from Ingersoll et al., to assess expression of LRRK2 mRNA by CD14^++^CD16^−^ and CD14^+^CD16^+^ monocytes subpopulations.** Differential gene expression was performed between CD14^++^CD16^−^ and CD14^+^CD16^+^ purified monocytes (Ingersoll et al., Comparison of gene expression profile between human and mouse monocyte subsets, Blood (2010) 115:e10-e19). Re-analysis of the expression data published in Gene Expression Omnibus (GEO) databank, accession GSE 18565 (human), revealed that LRRK2 and LRRK1 mRNA are not differentially expressed between CD14^++^CD16^−^ and CD14^+^CD16^+^ monocytes.
(TIF)

Figure S12 **LRRK2 but not LRRK1 promoter has extended 5′ end.** LRRK1 and -2 display similar intron-exon organization. However, LRRK1 is considerably shorter at the 5′ end.
(TIF)

Figure S13 **LRRK2 promoter has multiple 5′ potential GAS/ISRE sites.** IFN-γ inducible transcription factor ICSBP/IRF8 binds a set of DNA binding sites. Among these, ISRE and ICS contain the IRF recognition sequence (IRS), **AANNGAAA**, to which the DBD of the IRF family binds (see J. Interferon Cytokine Res. 22:145–152 (2002) for a review). Transcriptional activity further depends on the context and presence of additional proteins. Analysis of the LRRK2 proximal promoter reveals that five IRS such **AANNGAAA** (red underlined) consensus sequences are present.
(TIF)

Figure S14 **LRRK2 but not LRRK1 promoter has multiple 5′ potential GAS/ISRE sites.** The 2 kbp promoters show no significant sequence similarity (highlighted in green) suggesting divergent transcriptional regulation. In contrast to LRRK2 promoter, no **AANNGAAA** sequence is found in the proximal LRRK1 region. While we have not established that these represent *bona fide* IRS elements it appears likely that at least a subset of these is involved in the observed IFN-γ -inducible LRRK2 expression, in contrast to LRRK1.
(TIF)
