# Supplementary figures and images for: Regulation of LRRK2 Expression Points to a Functional Role in Human Monocyte Maturation
Source: PLoS One. 2011 Jun 27;6(6):e21519. doi: 10.1371/journal.pone.0021519 (PMC3124520; doi:10.1371/journal.pone.0021519)

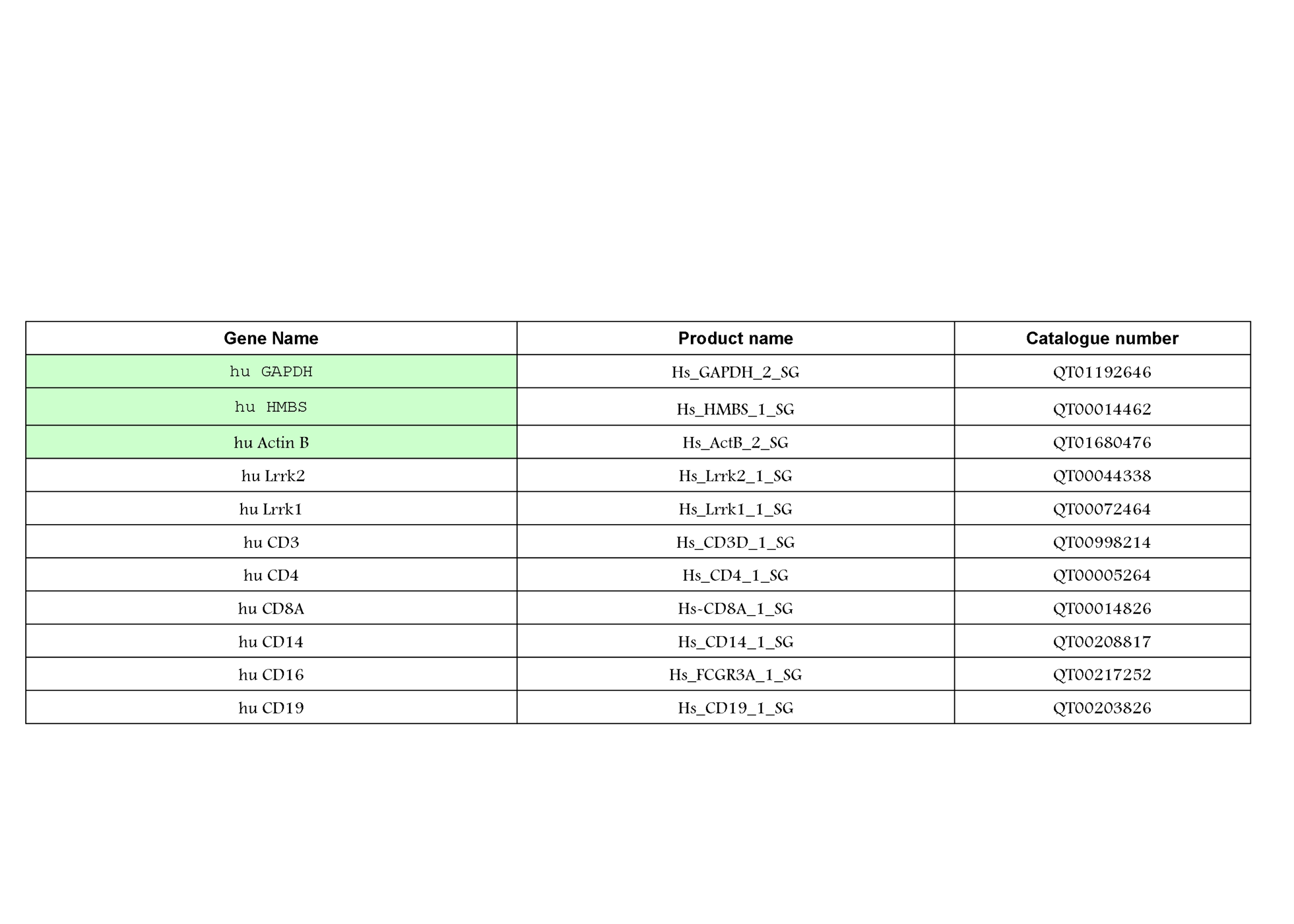

Supplement: Figure S1 — Reference of qPCR primers used in this study. Primers were obtained from QIAGEN. Glyceraldehyde 3-phosphate dehydrogenase (GAPDH), hydroxymethylbilane synthase (HMBS) and Actin B were used as internal control (house keeping genes). Leucine-rich repeat kinase 2 (LRRK2), Leucine-rich repeat kinase 1 (LRRK1), cluster of differentiation -3 (CD3), -4 (CD4), -8a (CD8a), -14 (CD14), -16 (CD16) and -19 (CD19). In all following experiments values of other genes were expressed as percentage of these three housekeeping genes. (TIF) [file pone.0021519.s001.tif]

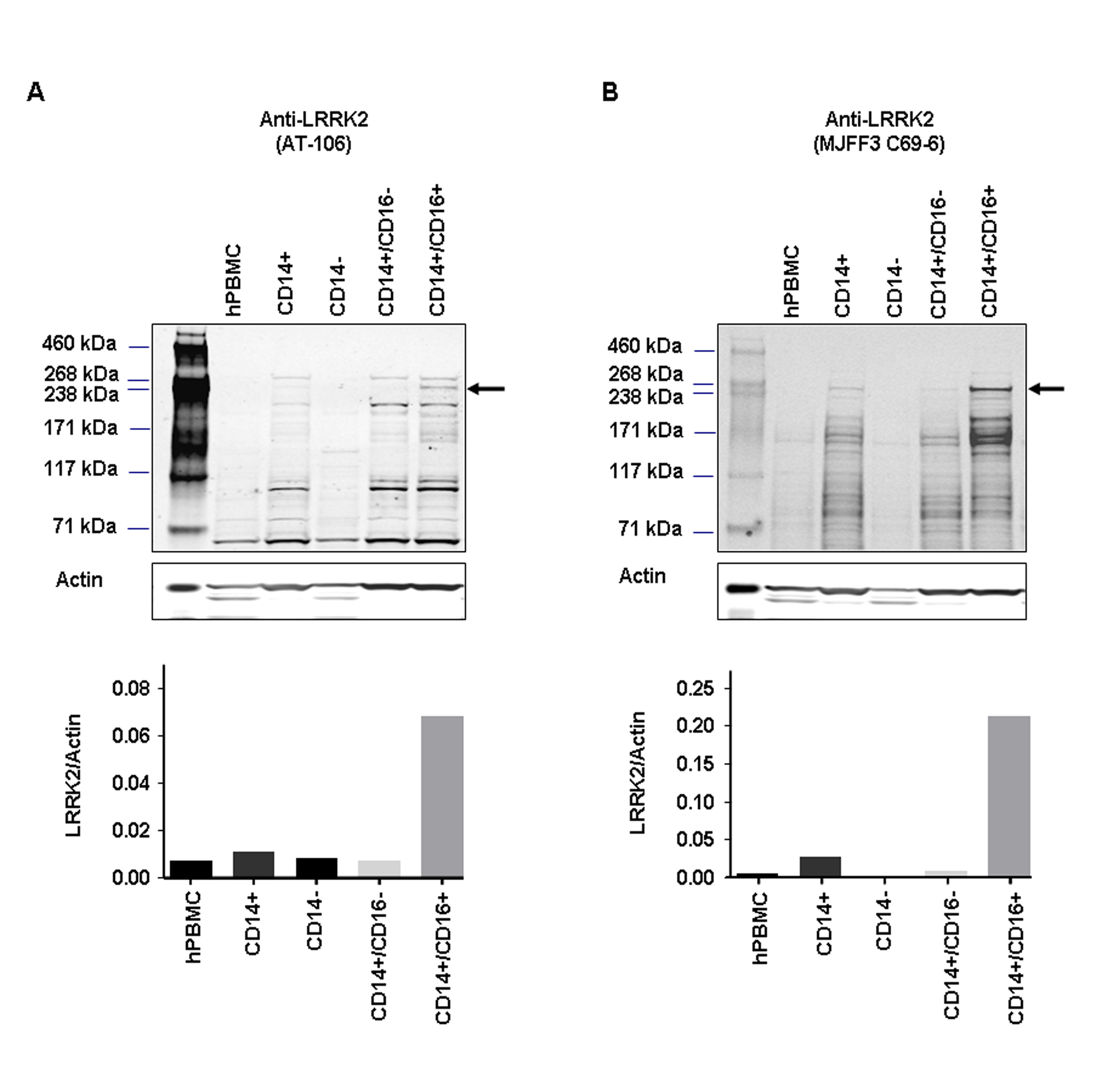

Supplement: Figure S2 — Validation of data obtained with the LRRK2 antibody ab60937 from Abcam using two additional LRRK2 antibodies. Experiments performed with AT106 from Alexis Biochemicals (panel A) or with MJFF3-c69-6 from Epitomics Inc. (panel B), confirmed the higher LRRK2 protein content in CD14+CD16+ monocytes as compare to CD14+CD16− cells. Western blots were performed the same day from the same material. Monocytes sub-populations were purified from hPBMC using immunomagnetic beads and all fractions including starting material (hPBMC) were loaded on the gel. CD14+ and CD14- respectively refer to monocytes and other cell types that are not expressing CD14. LRRK2 immunoreactive band is highlighted by the arrow. LRRK2 protein quantification was performed using the LI-COR Odyssey® and results are expressed in function of actin. Note that AT106 is also giving an additional band above LRRK2. As this band was not found using ab60937 or MJFF3-c69-6 antibodies, we did not consider it for LRRK2 quantification. (TIF) [file pone.0021519.s002.tif]

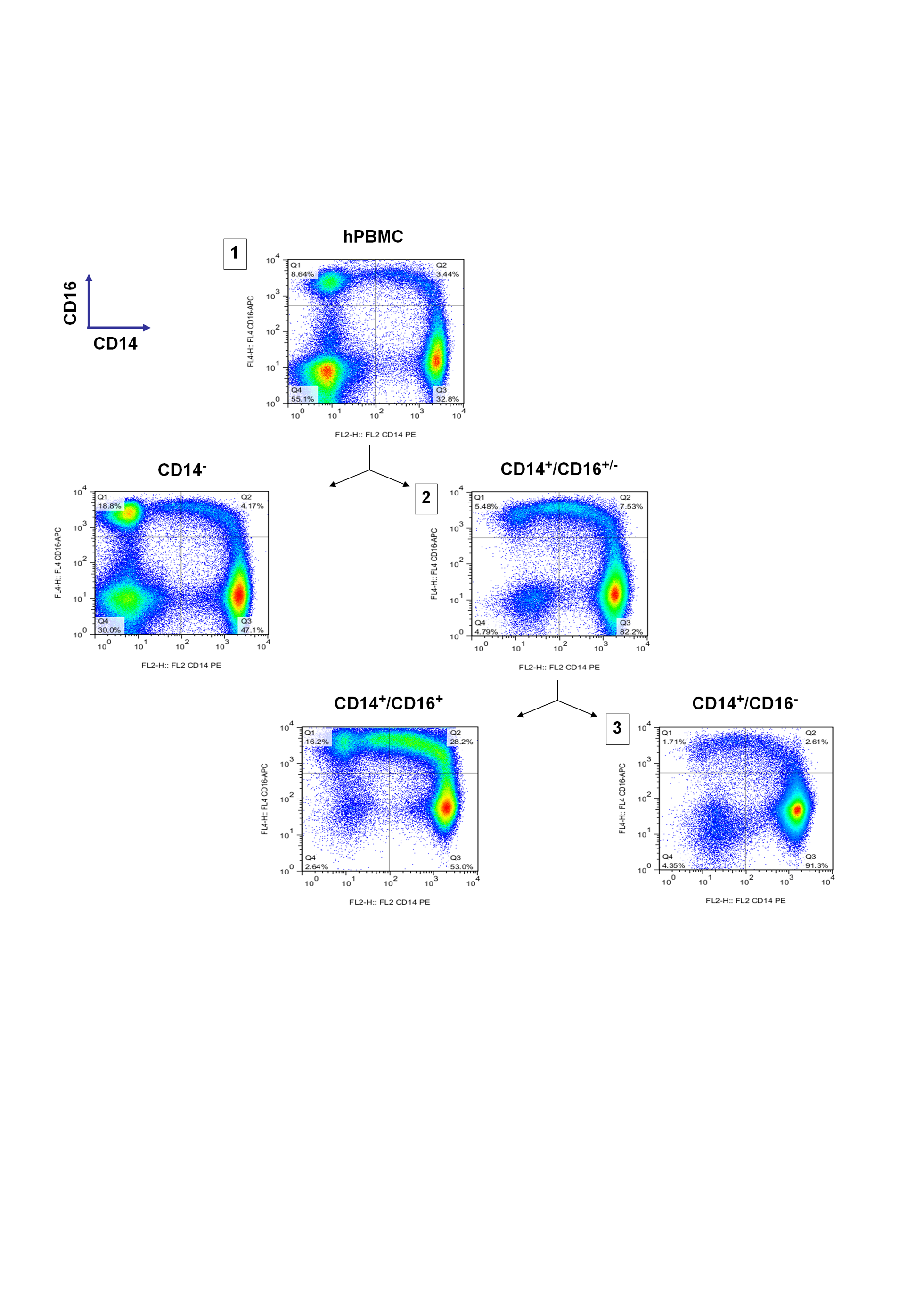

Supplement: Figure S3 — FACS assessment of CD14+ and CD14 + CD16+ enrichment from hPBMCs. FACS sorting of hPBMC sub-populations showing consecutives enrichment in CD14+ and CD14+/CD16+ monocytes. (1) At the beginning total PBMCs contained vast majority of CD14−/low cells corresponding to T-lymphocytes (bottom left quadrant) and NK cells (top left quadrant). (2) CD14+ enrichment using negative selection discarded most of T and NK cells, leaving a CD14+CD16+ and CD14+/CD16− mixed population. (3) This mixed population could be further worked on to purify CD14+CD16− sub-population to homogeneity or to enrich CD14+CD16+ cells using CD16+ positive selection. (TIF) [file pone.0021519.s003.tif]

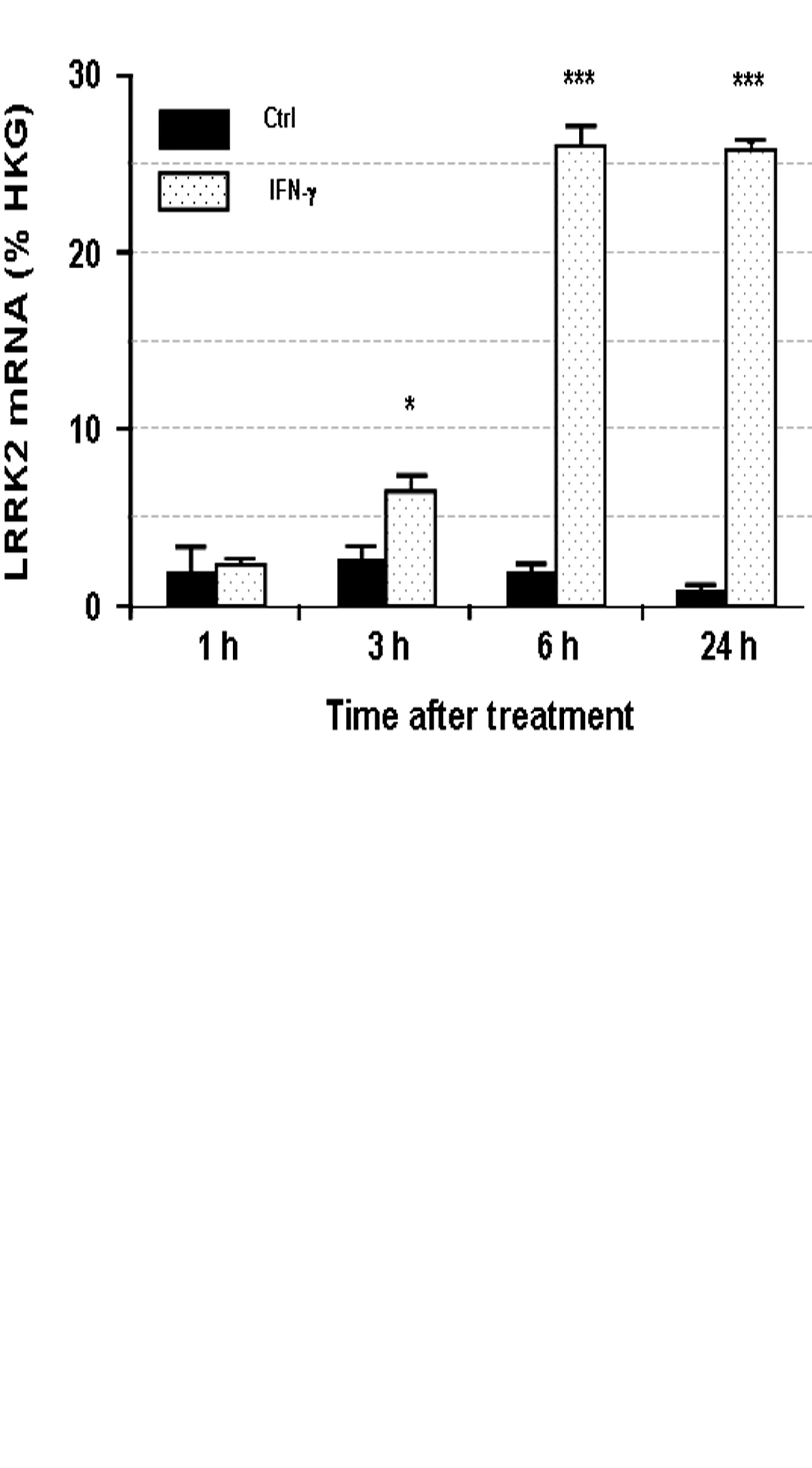

Supplement: Figure S4 — Time-course expression of LRRK2 by hPBMC stimulated by IFN-γ. At different times with or without IFN-γ (30 IU/mL) treatment cells were collected and LRRK2 mRNA expression quantified by qPCR. Increased expression was compared to time matching control without IFN-γ. (* p<0.05, ** p<0.01, *** p<0.005, Student t-Test, n = 3 for each point). (TIF) [file pone.0021519.s004.tif]

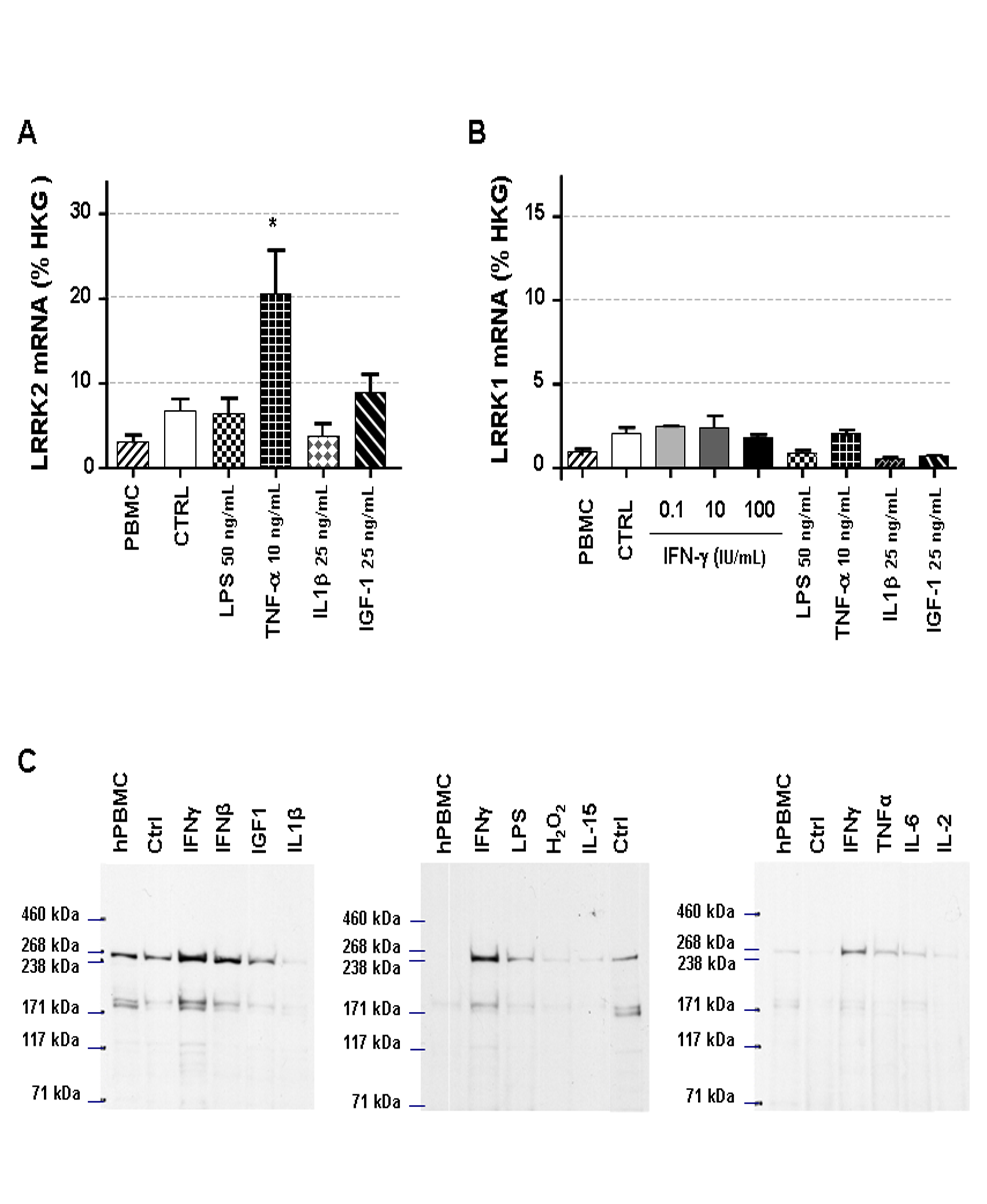

Supplement: Figure S5 — Effect of various cytokines on LRRK2 expression by hPBMCs. (A) and (B) Effect of various cytokines on LRRK2 and LRRK1 mRNA expression by hPBMC was monitored after 24 h of culture. LRRK2 protein content was also assessed (C). IFN-γ (0.1, 10 and 100 IU/mL); LPS (50 ng/mL), TNF-α (10 ng/mL), IGF-1 (25 ng/mL), IL-1β (25 ng/mL), IFN-β (100 IU/mL), IL-2 (10 ng/mL), IL-6 (10 ng/mL), IL-15 10 ng/mL and H2O2 (30 µM) were added at plating time. PBMC refers to freshly purified PBMC (starting material) and control refers to untreated hPBMC after one day in vitro. Western blot are representative of at least two independent experiments (except for IL-2, Cf. Table-I). mRNA values are expressed as percent of house keeping genes (%HKG). Statistical difference in normalized LRRK2 or LRRK1 mRNA amounts were calculated against values obtained on freshly isolated cells (* p<0.05; ** p<0.01, *** p<0.005, One Way Anova, with Bonferroni post-test; n = 3 for each point). (TIF) [file pone.0021519.s005.tif]

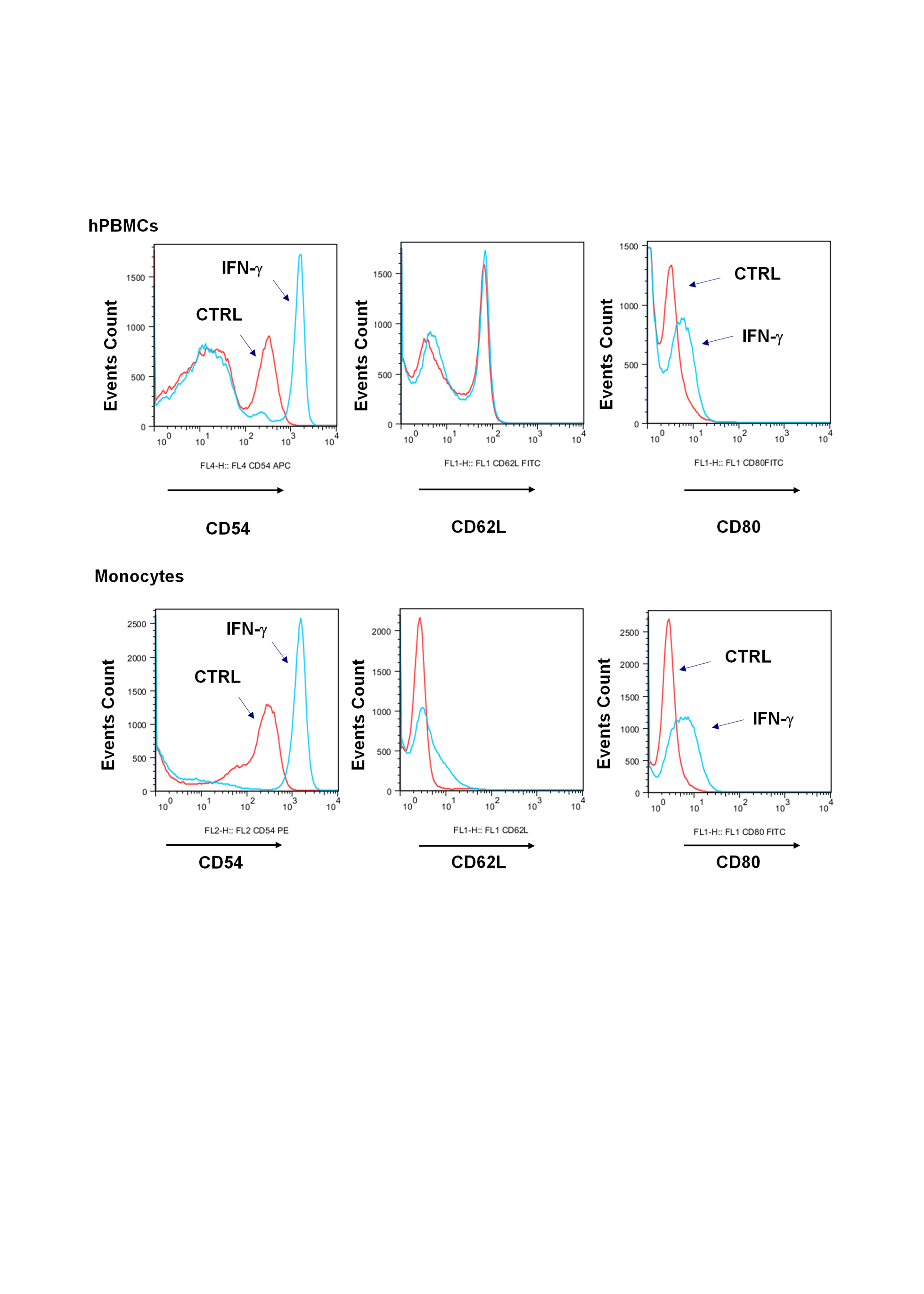

Supplement: Figure S6 — Assessment of monocytes activation by IFN-γ treatment of hPBMCs. Twenty four hours after in vitro treatment with IFN-γ (30 IU/mL), total PBMC (top panel) or purified monocytes (bottom panel) were harvested and analysed by FACS for various activation markers. Graph represents the number of cells (Events count) in function of staining intensity (Fluorescence Intensity). The shift towards high level of I-CAM (CD54), a molecule involved in cell adhesion, is characteristic of monocyte activation in response to IFN-γ. Note the similarity between the shift in PBMC and in purified monocytes populations. As lymphocytes also express CD54 another cell adhesion molecule L-selectin (CD62L) was measured. The absence of CD62L shift in response to IFN-γ and the low expression level of CD62L by monocytes confirmed that CD54 increase was due to monocytes activation. Finally, we studied the expression of a co-stimulatory signal ligand for T cells (CD80) expressed on B-cells and monocytes once activated. In PBMC as on purified monocytes we detected a slight shift in CD80 expression. CD80 expression will further increase with former maturation of monocytes. (TIF) [file pone.0021519.s006.tif]

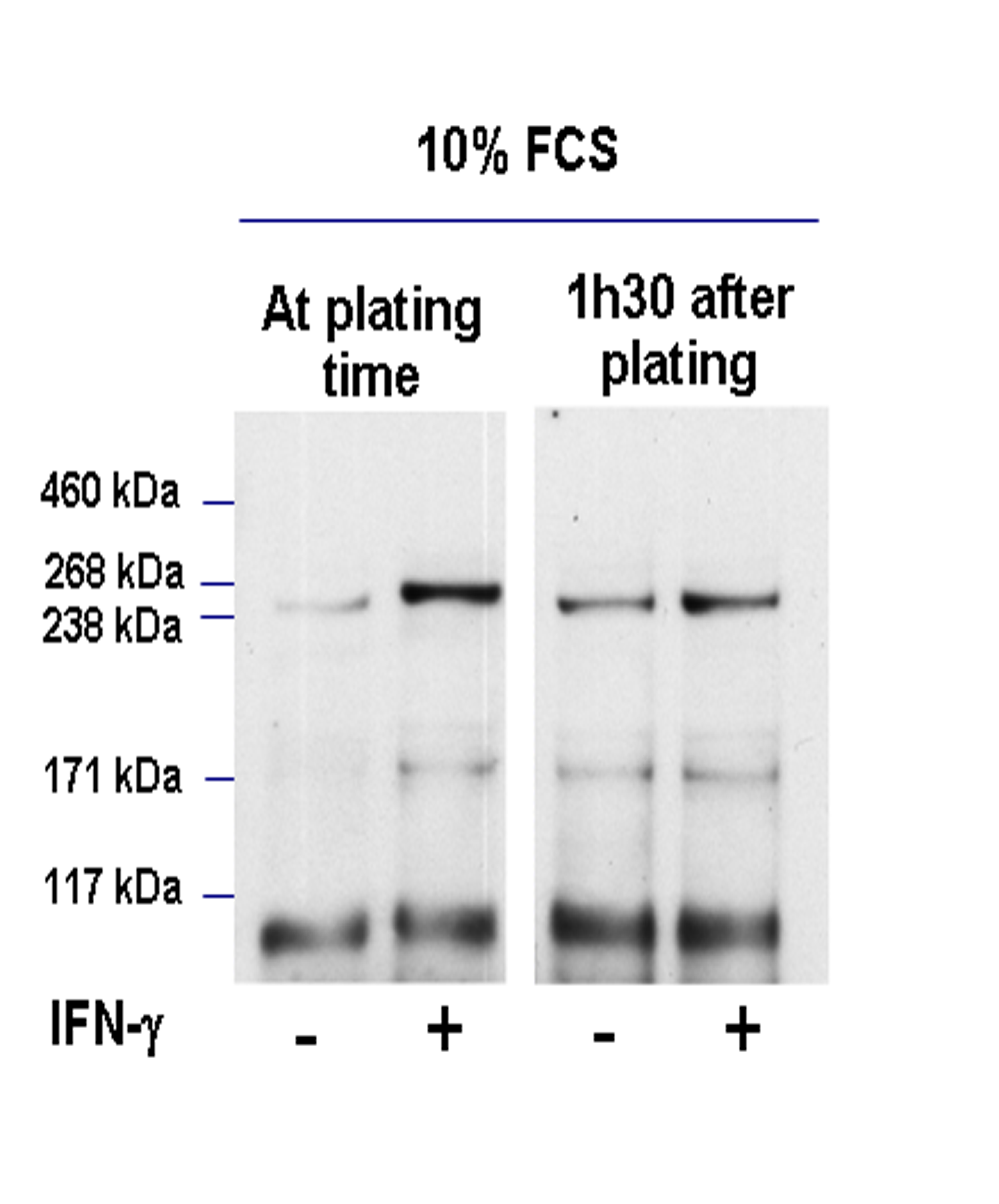

Supplement: Figure S7 — Serum free conditions induced LRRK2 expression by hPBMCs. PBMCs were plated in presence or absence of 10% foetal calf serum. Ninety minutes later medium was complemented with 10% FCS. Cultures were thereafter treated or not with IFN-γ (30 IU/mL) and cells collected 24 h later. LRRK2 protein levels were determined by western blot. Transient plating in serum free conditions induced LRRK2 expression but did not blunt IFN-γ response. Representative western blot of three independent experiments is shown. (TIF) [file pone.0021519.s007.tif]

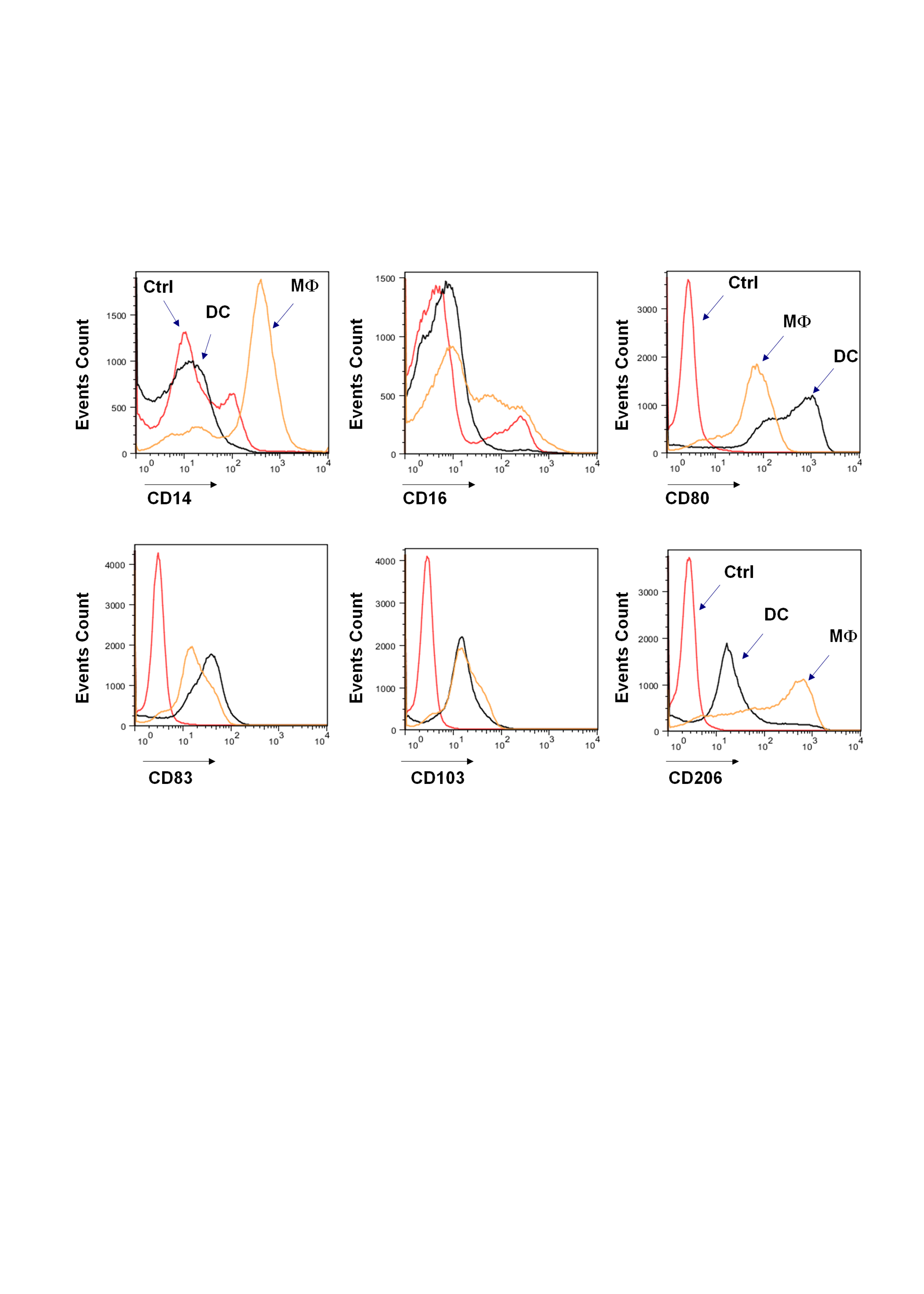

Supplement: Figure S8 — FACS analysis of monocytes maturation towards DC and macrophages. Purified monocytes CD14+CD16+/− populations were matured for 7 days in vitro with IL-4/GM-CSF towards dendritic cells (DC) or with LPS towards macrophages (MФ) phenotypes. DC and MФ are very heterogeneous in term of cell surface markers expression. As expected after 7 days of in vitro maturation, LPS treated monocytes have a MФ like pattern (brown line) with CD14High, CD16+, CD80+, CD83+, CD103+ and CD206High meanwhile IL-4/GM-CSF treated monocytes are DC like (black line) with a CD14Low, CD16Low, CD80High, CD83+, CD103+ and CD206+ pattern. The initial pattern obtained with purified monocytes one day after plating (red line) is included to highlight the switch in maturation markers expression. (TIF) [file pone.0021519.s008.tif]

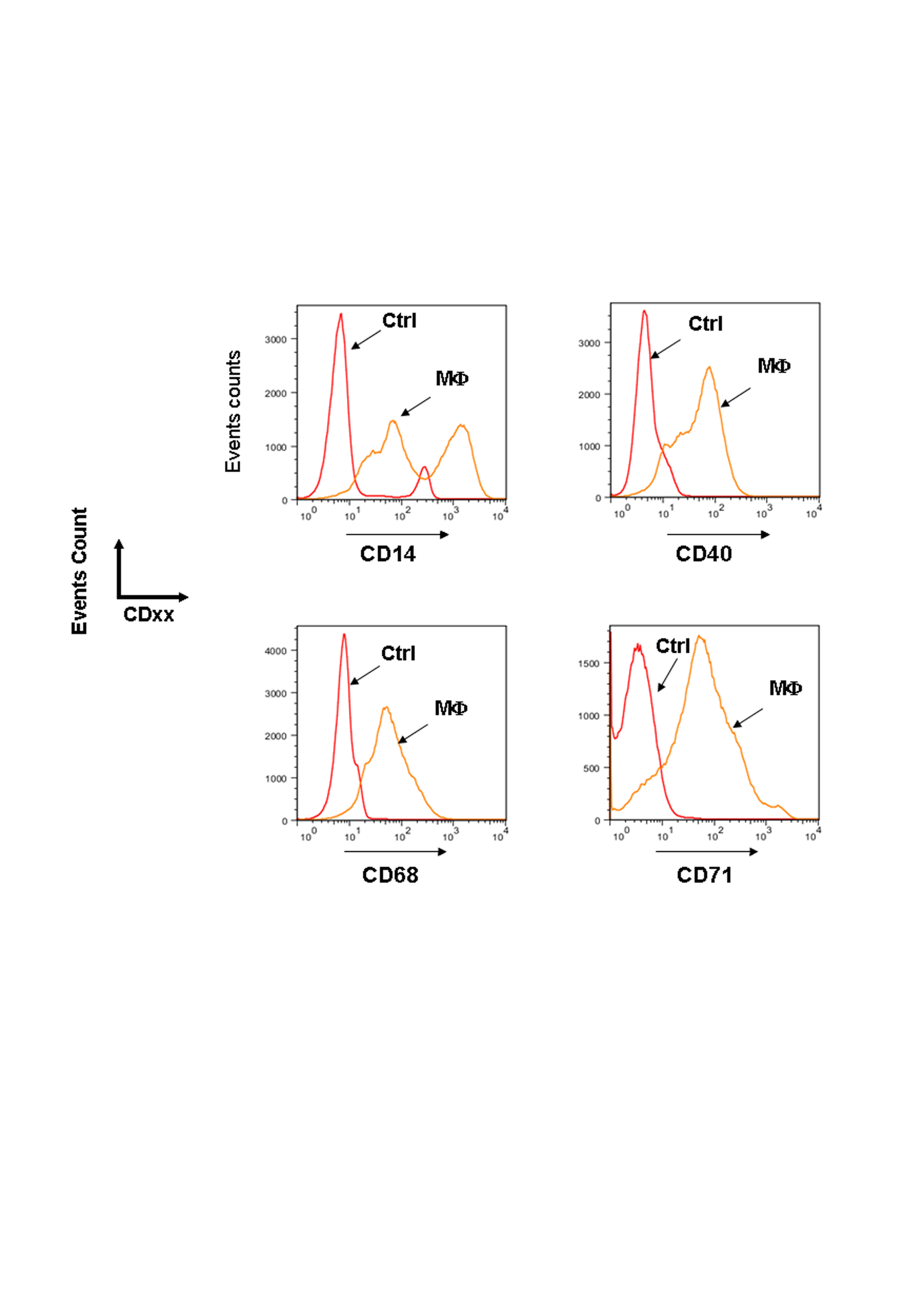

Supplement: Figure S9 — FACS analysis of monocytes maturation towards macrophages. To further document the induction of macrophage phenotype by LPS, additional macrophage markers, namely CD40, CD68 and CD71 were tested. After 7 days in vitro with LPS, monocytes CD14+CD16+/− populations matured towards macrophages (MФ) phenotypes with elevated expression of CD14 High, CD40+, CD68+ and CD71+ (orange line). The initial pattern obtained with purified monocytes one day after plating (red line) is included to highlight the switch in maturation markers expression. (TIF) [file pone.0021519.s009.tif]

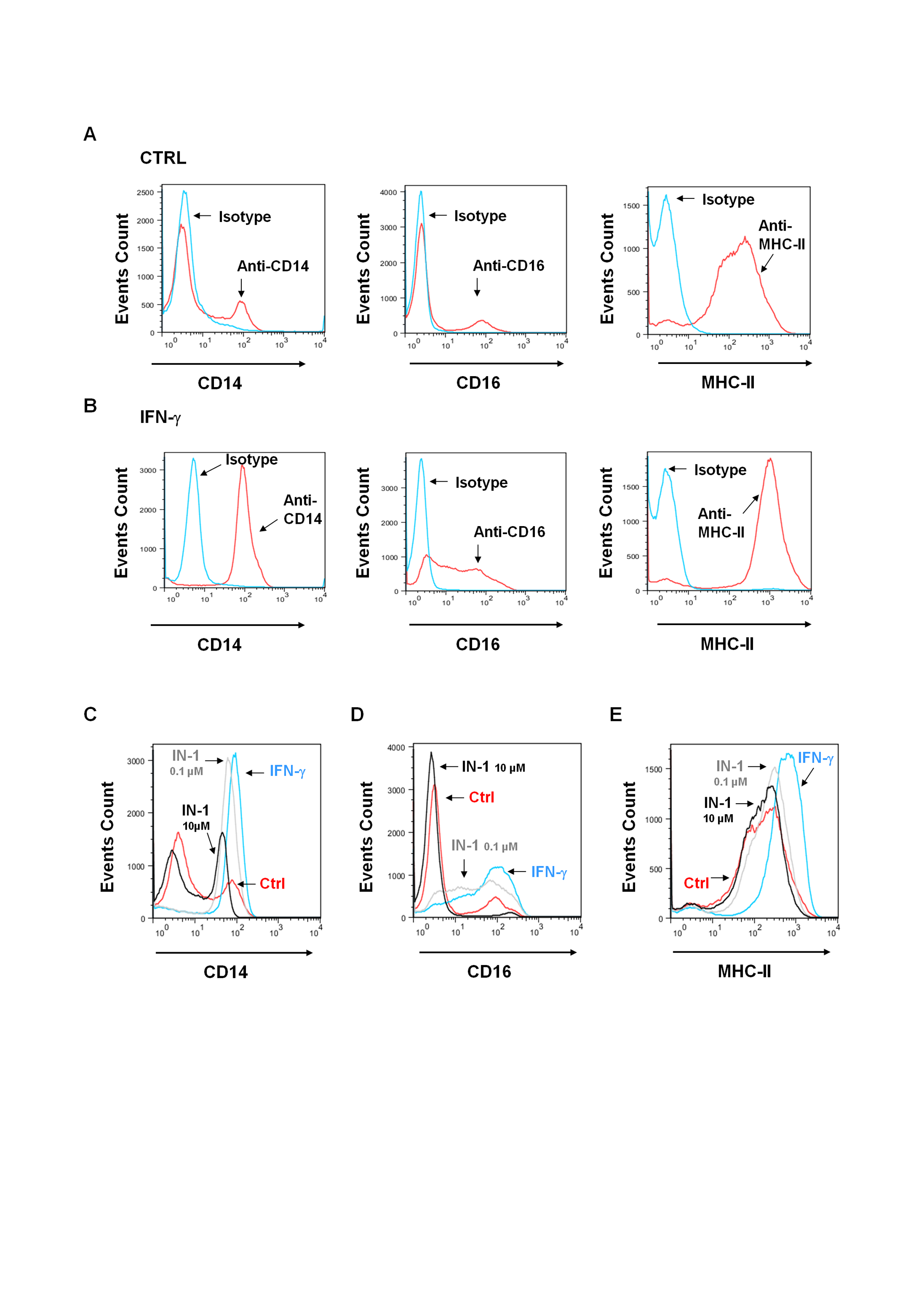

Supplement: Figure S10 — Effect of LRRK2 inhibitors on IFN-γ-induced cell surface markers by monocytes. After background determination using non-labeled cells and cells labeled with control isotypes, the threshold was set-up on IFN-γ (B) versus untreated (A) cells. With an average fluorescence value of 1.10+2, IFN-γ treatment (light blue line) induced a very strong shift in CD14+ (C) and CD16+ (D) expression as compare to untreated monocytes (red line). Due to endogenous high expression, MHC-II shift in response to IFN-γ was less obvious but still significant (E). Effects of 10 µM (dark line) and 0.1 µM (light grey) of IN-1 on CD14, CD16 and MHC-II expression were presented. At 10 µM IN-1 blocked IFN-γ -induced CD14, CD16 and MHC-II expression (p<0.005, p<0.005 and p<0.05, respectively. One-way-ANOVA, Bonferroni post-hoc test, n = 3 for each). (TIF) [file pone.0021519.s010.tif]

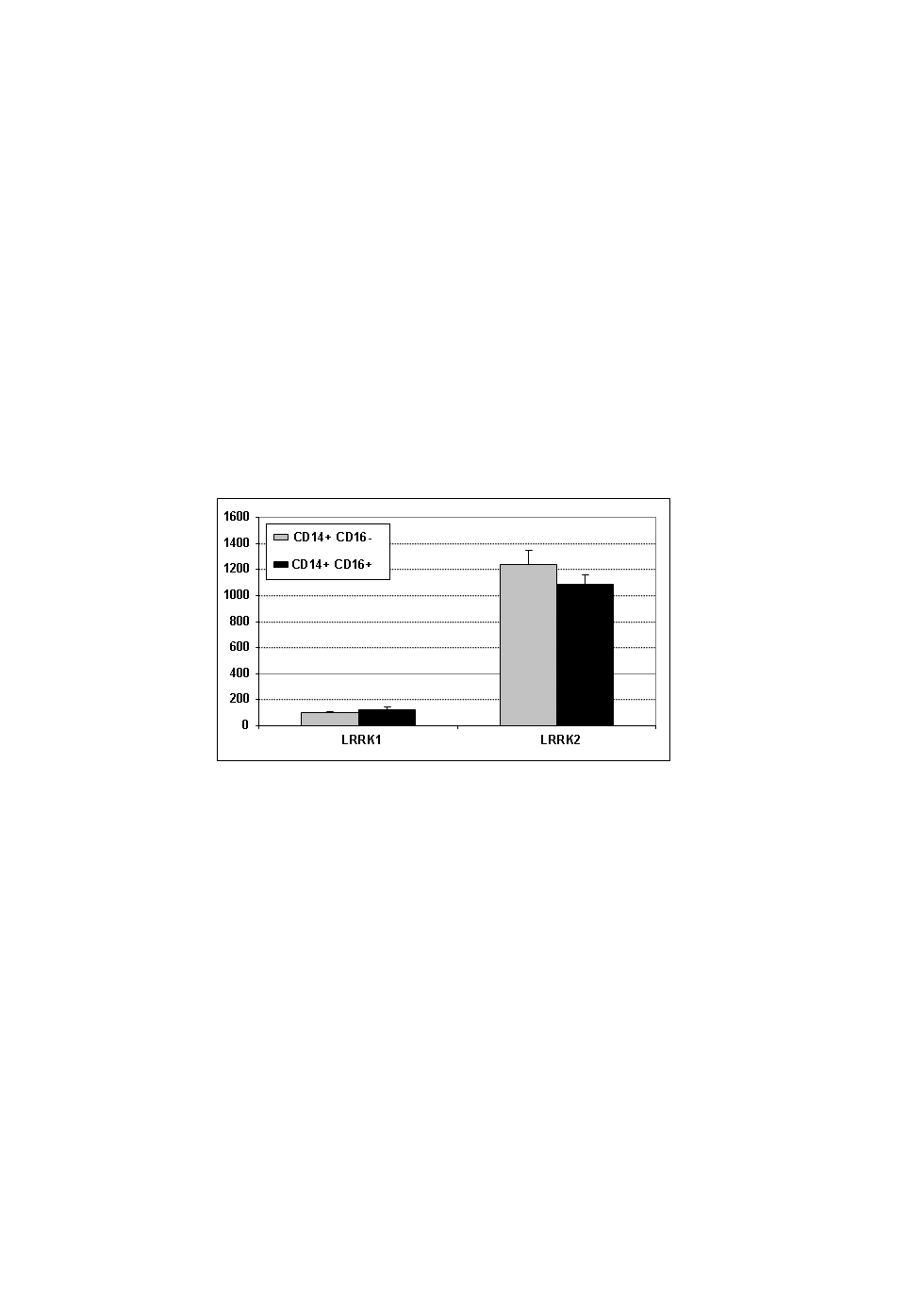

Supplement: Figure S11 — Analysis of transcriptome data from Ingersoll et al., to assess expression of LRRK2 mRNA by CD14++CD16− and CD14+CD16+ monocytes subpopulations. Differential gene expression was performed between CD14++CD16− and CD14+CD16+ purified monocytes (Ingersoll et al., Comparison of gene expression profile between human and mouse monocyte subsets, Blood (2010) 115:e10-e19). Re-analysis of the expression data published in Gene Expression Omnibus (GEO) databank, accession GSE 18565 (human), revealed that LRRK2 and LRRK1 mRNA are not differentially expressed between CD14++CD16− and CD14+CD16+ monocytes. (TIF) [file pone.0021519.s011.tif]

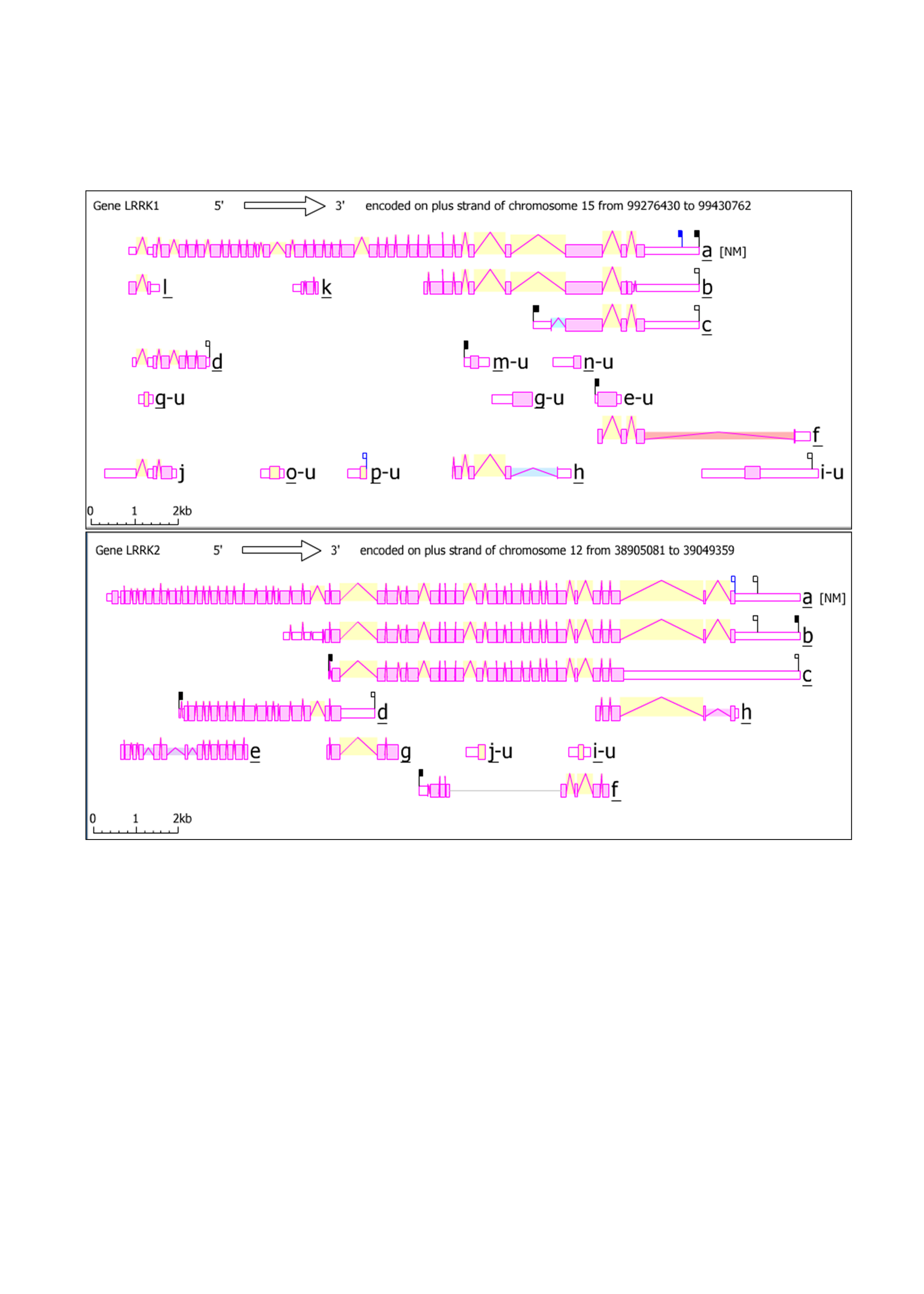

Supplement: Figure S12 — LRRK2 but not LRRK1 promoter has extended 5′ end. LRRK1 and -2 display similar intron-exon organization. However, LRRK1 is considerably shorter at the 5′ end. (TIF) [file pone.0021519.s012.tif]

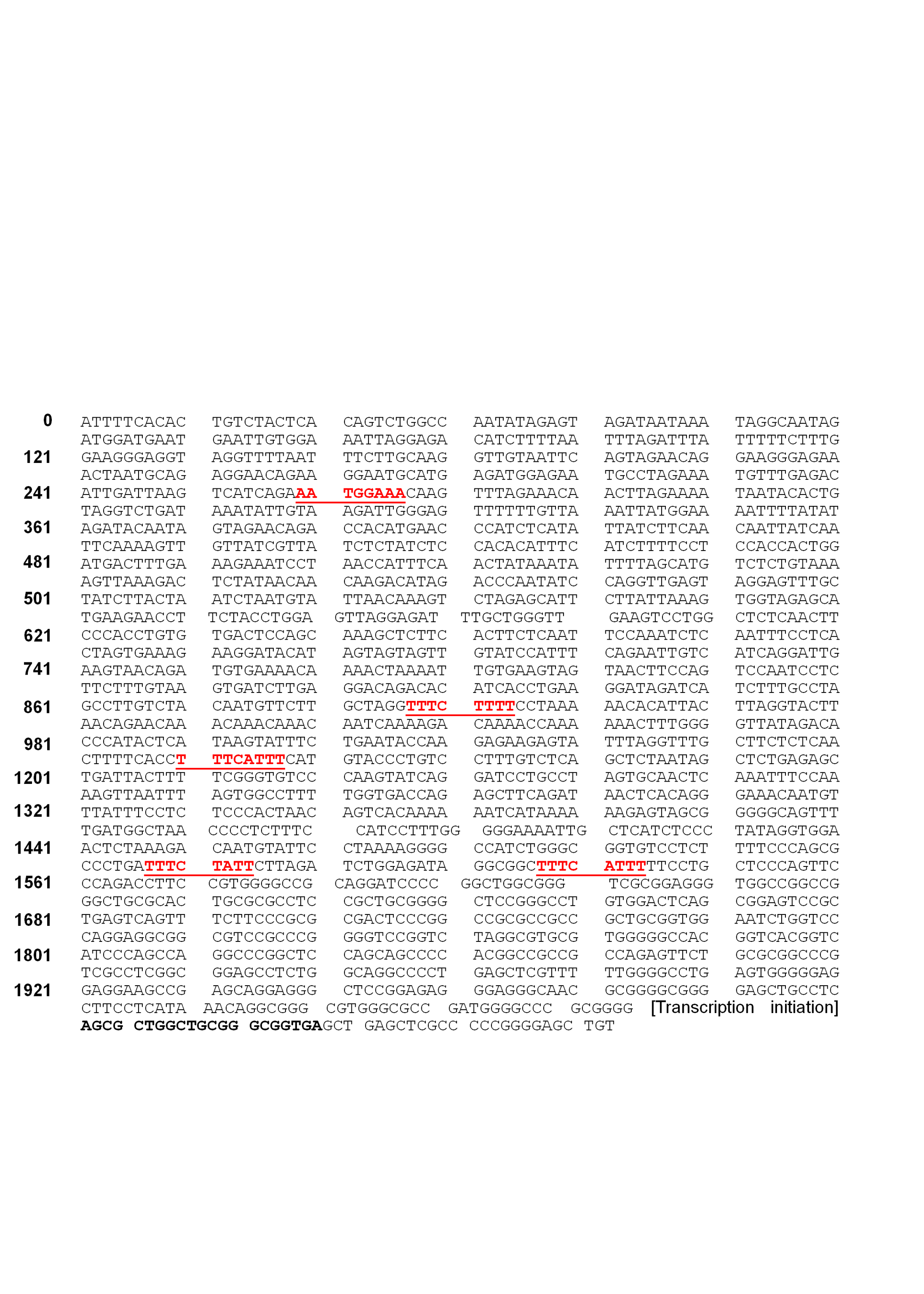

Supplement: Figure S13 — LRRK2 promoter has multiple 5′ potential GAS/ISRE sites. IFN-γ inducible transcription factor ICSBP/IRF8 binds a set of DNA binding sites. Among these, ISRE and ICS contain the IRF recognition sequence (IRS), AANNGAAA, to which the DBD of the IRF family binds (see J. Interferon Cytokine Res. 22:145–152 (2002) for a review). Transcriptional activity further depends on the context and presence of additional proteins. Analysis of the LRRK2 proximal promoter reveals that five IRS such AANNGAAA (red underlined) consensus sequences are present. (TIF) [file pone.0021519.s013.tif]

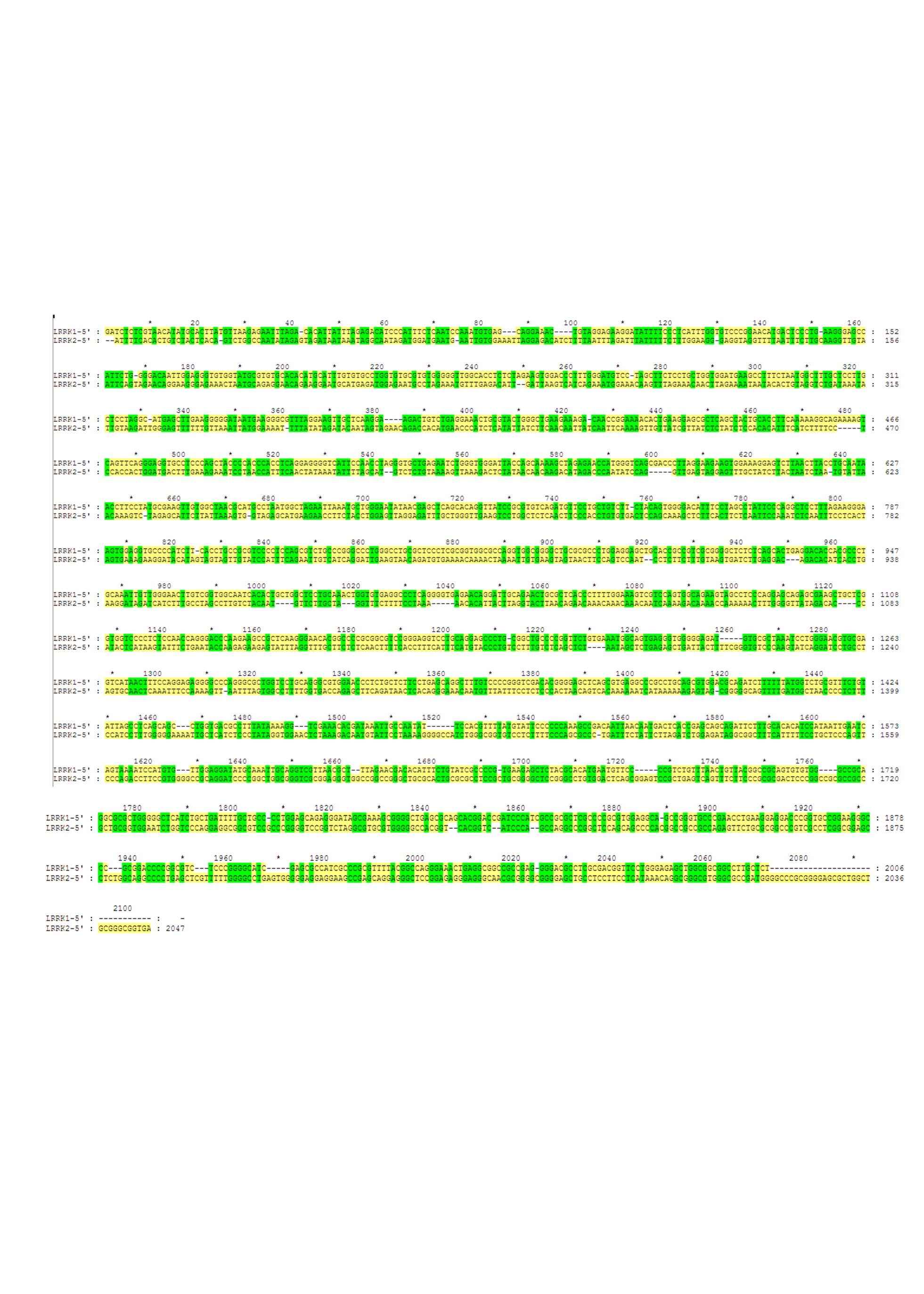

Supplement: Figure S14 — LRRK2 but not LRRK1 promoter has multiple 5′ potential GAS/ISRE sites. The 2 kbp promoters show no significant sequence similarity (highlighted in green) suggesting divergent transcriptional regulation. In contrast to LRRK2 promoter, no AANNGAAA sequence is found in the proximal LRRK1 region. While we have not established that these represent bona fide IRS elements it appears likely that at least a subset of these is involved in the observed IFN-γ -inducible LRRK2 expression, in contrast to LRRK1. (TIF) [file pone.0021519.s014.tif]
